# Microglial Deletion of *Hrh4* Alleviates Alzheimer's Disease Pathologies by Enhancing Microglial Phagocytosis of Amyloid‐β and Tau

**DOI:** 10.1002/advs.202505421

**Published:** 2025-10-24

**Authors:** Yi‐Jun Xu, Tan Wu, Larry Tso‐Lun Lo, Chi‐Chiu Ko, Xin Wang, Chi Him Eddie Ma

**Affiliations:** ^1^ Department of Neuroscience City University of Hong Kong Tat Chee Avenue Hong Kong SAR China; ^2^ Department of Surgery The Chinese University of Hong Kong Hong Kong China; ^3^ Department of Biomedical Sciences City University of Hong Kong Tat Chee Avenue Hong Kong SAR China; ^4^ Department of Chemistry City University of Hong Kong Tat Chee Avenue Hong Kong SAR China

**Keywords:** Alzheimer's disease, cAMP/TGF‐β1/Smad3, histamine receptor, in silico small‐molecule bioinformatics analysis, low‐dose ionizing radiation, microglial phagocytosis, VUF6002

## Abstract

Amyloid‐beta (Aβ) and hyperphosphorylated tau (p‐tau) aggregation are hallmark pathogenic events in Alzheimer's disease (AD). Microglial clearance of these toxic aggregates is essential, yet the underlying mechanisms remain poorly understood. This study demonstrates that low‐dose ionizing radiation (LDIR) provides protection against Aβ toxicity in vitro and rescues cognitive deficits in sporadic, young, and aged familial AD mouse models, including reductions in Aβ plaque, tauopathy, and microgliosis, while promoting microglial phagocytosis in aged 3xTg‐AD mice. Transcriptomic analysis identifies VUF6002, a histamine H4 receptor (H_4_R) antagonist, which mimics the beneficial effects of LDIR by promoting microglial activity. VUF6002 treatment restores cognitive function in aged 3xTg‐AD and APPswe/PSEN1dE9 mice and significantly increases Aβ and p‐tau clearance by resident microglia. Mechanistically, deletion of *Hrh4* in microglia, but not in neurons, reverses cognitive deficits and mitigates key AD pathogenesis by activating the cAMP/TGF‐β1/Smad3 pathway. These beneficial effects are completely abolished by inhibition of TGF‐β receptor 1 signaling, which is also downregulated in AD patients. Collectively, these findings reveal a H_4_R/cAMP/TGF‐β1/Smad3 signaling axis involved in microglial phagocytosis and cognitive function, serving as a novel therapeutic target for AD.

## Graphical abstract

Histamine H4 receptor (H4R) antagonist VUF6002 mimics low‐dose X‐ray irradiation in aged Alzheimer's disease (AD) mice, enhancing microglial clearance of amyloid‐beta/hyperphosphorylated tau aggregates and restoring cognition. Microglial H4R deletion activates cAMP/TGF‐β1/Smad3 pathway, enhancing phagocytosis, while TGF‐β receptor 1 deletion abolishes these effects. Impaired TGF‐β1 signaling is observed in AD patients, highlighting cAMP/TGF‐β1/Smad3 pathway as a promising therapeutic target.

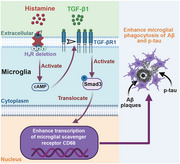

## 1 Introduction

Alzheimer's disease (AD) is the most prevalent form of dementia, characterized by a progressive decline in cognitive function. Key pathological features of AD include extracellular amyloid‐β (Aβ) aggregation, intracellular neurofibrillary tangles formation primarily caused by hyperphosphorylated tau (p‐tau) aggregation, reactive microgliosis, and neuroinflammation.^[^ ^1^, ^2^, ^3^ ^]^ Genome‐wide association studies have identified over 20 genetic risk factors linked to the development of late‐onset AD. Among these, genes such as *TREM2*, *CR1*, *CD33*, *MS4A*, *CLU*, *ABCA7*, *HLA‐DRB5/HLA‐DRB1*, *INPP5D*, and *APOE* are particularly implicated in modulating microglial reactivity and inflammatory responses.^[^ ^4^, ^5^, ^6^ ^]^ Furthermore, a retrospective analysis of 130 AD patients demonstrated a strong association between microglia and both Aβ and tau pathology, indicating that microglial response plays a critical role in the disease's pathological progression.^[^ ^7^, ^8^ ^]^ Microglia function as immune sentinel cells in the brain, playing a key role in the phagocytic clearance of various insults, including Aβ and p‐tau aggregates, myelin debris, and apoptotic cells.^[^ ^1^, ^9^, ^10^ ^]^ They can reorganize less‐dense Aβ plaques into more compact core‐dense plaques, which are subsequently phagocytosed and degraded within microglial lysosomes.^[^ ^11^, ^12^, ^13^ ^]^ However, during aging, intracellular lipid accumulation and chronic inflammation impair microglial phagocytic capacity, thereby promoting the accumulation of pathological markers associated with AD.^[^ ^10^, ^14^, ^15^, ^16^ ^]^ Recent advances have demonstrated that modulating microglial genes such as *TREM2*,^[^ ^17^ ^]^ *SYK*,^[^ ^11^ ^]^ *CD33*,^[^ ^18^ ^]^ *DAP12*,^[^ ^19^ ^]^ *PLCG2*,^[^ ^20^ ^]^ *ABI3*,^[^ ^21^ ^]^ and *HCAR2* ^[^ ^22^ ^]^ through small molecules or genetic interventions can enhance Aβ phagocytosis by microglia, potentially mitigating AD pathogenesis in mouse models. The identification of novel microglial genes and small molecules that facilitate Aβ clearance could serve as a therapeutic approach for AD, offering new avenues for intervention and disease modification.^[^ ^11^, ^17^ ^]^

Growing evidence suggests that low‐dose ionizing radiation (LDIR) induces a beneficial adaptive response, enhancing both the overall functional capacity and immune responses of organisms.^[^ ^23^, ^24^ ^]^ The beneficial effects of LDIR (e.g., X‐ray and γ‐ray) have been well‐documented, including stimulation of cell growth, extension of average lifespan, and neuroprotection in various animal disease models, as well as beneficial effects on human inflammatory and leukemia diseases.^[^ ^25^, ^26^, ^27^ ^]^ In AD patients, a case report of an 81 years old female with advanced AD demonstrated the significant benefits of LDIR exposure after undergoing five computed tomography (CT) scans, with a total dose of 207 mGy. Remarkably, she regained her cognitive function, speech, movement, appetite, and responsiveness just two days after the first two CT scans, with a total dose of 82 mGy, leading to notable enhancements in her quality of life.^[^ ^28^ ^]^ A subsequent clinical study showed that three out of four patients with advanced AD treated with LDIR through CT scans exhibited improvements in cognitive function and behavior within just one day.^[^ ^29^ ^]^ However, LDIR therapy remains controversial, as it is widely recognized that repeated exposure to ionizing radiation could potentially increase the risk of cancer. Animal studies demonstrated that relatively high‐dose X‐ray irradiation (9–10 Gy) reduced Aβ deposition, decreased neuronal loss, and enhanced cognitive function in the 5xFAD mouse model of AD.^[^ ^30^, ^31^, ^32^ ^]^ Eight weeks after fractionated X‐ray irradiation (5 Gy), there was a significant reduction in Aβ plaque deposition and cognitive decline in APPswe/PSEN1dE9 (APP/PS1) and 5xFAD mice,^[^ ^30^, ^31^ ^]^ while also suppressing both Aβ and phosphorylated tau pathologies in aged 3xTg‐AD mice.^[^ ^33^ ^]^ Chronic low‐dose γ‐ray exposure (0.1 mGy) was shown to reprogram neuroimmune responses in an *ApoE−/−* AD mouse model, which resulted in reduced hippocampal microglial density and TNF‐α‐driven neuroinflammation.^[^ ^34^ ^]^ Additionally, LDIR modulated cytokine profiles by downregulating proinflammatory TNF‐α and upregulating anti‐inflammatory TGF‐β in the 5xFAD mice.^[^ ^31^ ^]^ LDIR modulated microglial polarization by directly inducing a phenotypic shift in microglia from a proinflammatory to an anti‐inflammatory state within the brain.^[^ ^31^ ^]^ Notably, LDIR upregulated *Tmem2*, a marker of disease‐associated microglia (DAM) with enhanced Aβ phagocytic capacity, in LPS‐stimulated BV2 microglia.^[^ ^35^ ^]^ However, whether LDIR directly enhances microglial phagocytosis of Aβ remains unknown.

Our recent study demonstrated that LDIR promoted axon regeneration and functional recovery after sciatic nerve injury. Subsequent genome‐wide transcriptional and small‐molecule bioinformatics analyses identified a small molecule that mimicked the beneficial effect of LDIR.^[^ ^24^ ^]^ We therefore conclude that changes in gene expression profiles following LDIR contribute to adaptive responses in living organisms. While LDIR may represent a promising, albeit controversial, therapy for AD, there is still much to learn before it can be established as a routine, effective, and safe medical treatment. By identifying key gene signatures and small molecules associated with LDIR‐induced protective effects, we could develop safe and effective pharmacological therapies for AD.

In the current study, we first demonstrated the protective effects of low‐dose X‐ray irradiation on Aβ toxicity in neuronal cell culture. LDIR restored the cognitive deficits to normal control levels in three mouse models of AD (sporadic, young and aged familial AD mice) and alleviated key features of AD pathogenesis in 15 month old 3xTg‐AD mice by reducing Aβ plaque aggregation, tauopathy, and microgliosis, while also enhancing microglial phagocytosis. By performing a whole transcriptome analysis on the hippocampus from X‐ray‐irradiated 3xTg‐AD mice and their age‐matched sham‐irradiated mice, we identified a top‐ranked small molecules, VUF6002. VUF6002 is a potent and selective antagonist of the histamine H4 receptor (H_4_R, encoded by the *Hrh4* gene),^[^ ^36^, ^37^ ^]^ which reversed the cognitive deficits in streptozotocin (STZ)‐induced sporadic AD mice, as well as in young and aged familial AD mice. More importantly, VUF6002 treatment also reduced major AD pathogenesis in two aged AD mouse models. Fluorescence‐activated cell sorting (FACS) of resident microglia from VUF6002‐treated aged APP/PS1 mice showed a significant increase in the clearance of methoxy‐X04‐labeled Aβ compared to controls. Mechanistically, deletion of *Hrh4* in microglia, but not neurons, reversed cognitive deficits and decreased AD pathology in 8 months old APP/PS1 mice and two advanced AD mouse models, primarily through activation of the TGF‐β1/Smad3 signaling pathway. Further, microglial deletion of TGF‐β receptor 1 (TGF‐βR1) completely abolished the promoting effects of microglial *Hrh4* deletion on microglial phagocytosis and cognitive function recovery in aged APP/PS1 mice.

## 2 Results

### 2.1 LDIR Reverse Cognitive Function Deficits and Major AD Pathogenesis in Young and Aged AD Mice

To explore the protective effects of LDIR, we first assessed whether LDIR could protect Neuro‐2a cell from Aβ toxicity, since Aβ aggregation is considered one of the primary causes of AD. Treatment of Neuro‐2a cells for 48 h with increasing concentration of Aβ_1–42_ (10–40 µm) resulted in a significant cell death (20–32%)^[^ ^38^ ^]^ (Figure S1A, Supporting Information). LDIR at doses ranging from 100–400 mGy protected the cells (88–93%) from Aβ toxicity, with the effect reaching a plateau at 400 mGy (Figure S1B, Supporting Information), without affecting overall cell survival (Figure S1C, Supporting Information). We then extended these findings to in vivo studies to evaluate the recovery of learning and memory in both sporadic and familial mouse models of AD.

Next, we examined the beneficial effects of LDIR in a sporadic mouse model of AD by whole‐body X‐ray irradiation with a total accumulated dose of 400 mGy. A widely used sporadic AD mouse model is generated by intracerebroventricular injection of STZ, which indices severe learning and memory deficits.^[^ ^39^, ^40^ ^]^ The mice received X‐ray exposures at 100 mGy on days 0, 7, 14, and 21 following STZ injection (Figure S2A, Supporting Information). STZ mice showed impaired exploratory behavior and spatial memory, as evidenced by a reduced spontaneous alternation in the Y‐maze test. X‐ray treatment improved their alternation rate from 50% to 67%, approaching the 73% observed in irradiated sham controls (Figure S2B, Supporting Information). In terms of short‐term and recognition memory, assessed by the novel object recognition (NOR) test, STZ administration significantly reduced the recognition index to 40% compared to 69% in sham controls. However, X‐ray irradiated STZ mice exhibited a remarkable improvement in short‐term memory, with a recognition index of 70%, and spent a comparable amount of time exploring novel objects as the sham controls, which had a recognition index of 64% (Figure S2C, Supporting Information). The mice were further evaluated using the Barnes maze, a standard test for spatial learning and long‐term memory.^[^ ^41^ ^]^ STZ mice were tested daily over four consecutive days with three trials per day and video tracked to measure the time (latency) taken to find a hidden escape box. During training, latency remained consistently higher in the STZ mice than in the other mouse groups (Figure S2D, Supporting Information). In the probe test, where the escape box was removed, X‐ray irradiated STZ mice showed shorter latency to reach the target hole for the first time (Figure S2E, Supporting Information) and spent more time in the target quadrant compared to the untreated STZ mice (Figure S2F, Supporting Information), as shown by the tracking plot analysis (Figure S2G, Supporting Information).

The 3xTg‐AD mouse is a widely used and well‐characterized familial form of AD that develops amyloid plaques and neurofibrillary tangles in a specific temporal and spatial manner. Notably, these mice begin to show cognitive impairment at around 4 months of age, with a marked decline in spatial memory retention.^[^ ^42^, ^43^ ^]^ To evaluate the therapeutic potential of X‐ray irradiation, 9 months old 3xTg‐AD mice received biweekly X‐ray irradiation at 50 mGy for 3.5 months (total accumulated dose: 400 mGy) (Figure S3A, Supporting Information). This X‐ray irradiation treatment fully restored exploratory behavior and spatial memory in the Y‐maze (Figure S3B, Supporting Information), improved short‐term and recognition memory in the NOR (Figure S3C, Supporting Information), and enhanced long‐term spatial memory performance in the Barnes maze (Figure S3D–G, Supporting Information).

To test if LDIR could mitigate age‐related decline in advanced stage of AD, we treated 15 months old 3xTg‐AD mice with the same biweekly X‐ray protocol of 50 mGy over 3.5 months, resulting in a total cumulative dose of 400 mGy (Figure S4A, Supporting Information). LDIR treatment restored spatial recognition memory in these aged 3xTg‐AD mice to levels comparable with control or sham‐irradiated mice during the Y‐maze tests (Figure S4B, Supporting Information). Additionally, LDIR reversed deficits in short‐term recognition memory in the NOR test by increasing the proportion of exploration time spent on novel object (Figure S4C, Supporting Information). We further evaluated long‐term spatial memory using the Barnes maze; the latency to find the target hole was not significantly different between LDIR‐treated aged 3xTg‐AD mice and both the wildtype (WT) and sham‐irradiated WT controls over the 4 days training period (Figure S4D, Supporting Information). Moreover, LDIR‐treated aged 3xTg‐AD mice made significantly fewer errors before reaching the target hole (Figure S4E, Supporting Information) and spent more time in the target quadrant during the day 5 probe trial (Figure S4F,G, Supporting Information), compared to sham‐irradiated aged 3xTg‐AD mice. These results suggest that X‐ray treatment could prevent the progression of cognitive function deficits in STZ mice and reverse existing cognitive deficits in both young and aged 3xTg‐AD mice. No observable effects were seen in sham‐irradiated control mice (WT + 400 mGy), which underwent the same irradiation protocol, indicating the specific beneficial effects of LDIR treatment on cognitive functions in AD mice.

An increase in Aβ load accelerates disease progression and drives tau pathology, ultimately leading to neurodegeneration in AD.^[^ ^1^ ^]^ To determine whether the cognitive function improvements observed with LDIR were associated with the reductions in Aβ load, we performed immunostaining using 6E10 antibodies to visualize Aβ deposits in the subiculum area of 15 months old aged 3xTg‐AD mice after 3.5 months of X‐ray treatment (400 mGy). The total number of 6E10‐labeled Aβ plaques was significantly decreased (Figure S5A, Supporting Information). Enzyme‐linked immunosorbent assay (ELISA) is used to measure levels of soluble Aβ_1–40_ and Aβ_1–42_, which are considered more toxic than their insoluble counterparts.^[^ ^44^, ^45^ ^]^ In X‐ray‐irradiated aged 3xTg‐AD mice, Aβ_1–40_ levels were reduced by 59% in the hippocampus and 69% in the cortex (Figure S5B, Supporting Information). Similarly, Aβ_1–42_ levels decreased by 52% in the hippocampus and 43% in the cortex (Figure S5C, Supporting Information). Additionally, tau hyperphosphorylation was substantially decreased, with a 50% reduction observed via immunohistochemistry using the AT8 antibody (Figure S5D, Supporting Information) and Western blot analysis confirmed a 62% decrease (Figure S5E, Supporting Information). Microgliosis, a hallmark of AD pathology, was also significantly attenuated: the number of activated microglia in the subiculum was reduced by 53% (Figure S5F, Supporting Information), and microglial phagocytic activity increased by 74%, as demonstrated by colocalization of Iba1‐positive microglia with 6E10‐positive Aβ plaques^[^ ^46^, ^47^ ^]^ (Figure S5G, Supporting Information). Notably, WT mice that received the same X‐ray treatment showed no significant changes in these measures, indicating that the effects were specific to the AD mice.

### 2.2 In Silico Small‐Molecule Bioinformatics Analysis of LDIR Gene Expression Signature Identifies VUF6002 as a Therapeutic Small Molecule

**Figure 1:**
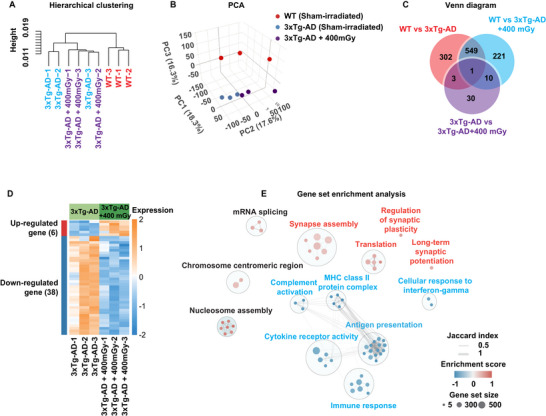
Whole transcriptome analysis identifies gene signatures of low‐dose X‐ray radiation (LDIR) for in silico small molecules screening. Hippocampus was harvested from sham‐irradiated wildtype (WT) mice, sham‐irradiated 3xTg‐AD mice, and LDIR‐treated 3xTg‐AD mice for whole transcriptome analysis using RNA sequencing (*n* = 3 per group). A,B) Hierarchical clustering and PCA plot revealed three distinct clusters, indicating that LDIR‐induced transcriptomic changes in 3xTg‐AD mice. C) Venne diagram illustrated the number of differentially expressed genes identified across the various group comparisons. D) LDIR‐induced distinct transcriptional signatures in 9 months old 3xTg‐AD mice, comprising 44 DEGs (6 upregulated and 38 downregulated). E) Gene Set Enrichment Analysis was conducted to characterize pathway‐level alterations mediated by LDIR in 3xTg‐AD mice, revealing functional annotations of the gene expression profiles.

Our results demonstrated that LDIR protected against Aβ toxicity and tau hyperphosphorylation in 3xTg‐AD mice, leading to improved cognitive function. However, systemic LDIR is rarely used clinically due to concerns about potential damage to sensitive tissues such as the hematopoietic system, reproductive organs, and ocular lenses. Pharmacological agents offer a safer alternative, providing flexible dosing, adjustable treatment schedules, and timing without the risks associated with systemic radiation. We hypothesized that small molecules associated with the gene regulatory network of LDIR, could also reverse cognitive function deficits in AD mice. To test this hypothesis, we performed a whole transcriptome analysis and small‐molecule bioinformatics analysis on hippocampi of 3xTg‐AD and their aged‐matched WT mice, 3.5 months following sham or 400 mGy X‐ray irradiation. Hierarchical clustering and principal component analysis clearly distinguished WT mice, 3xTg‐AD mice, and X‐ray‐irradiated 3xTg‐AD mice (**Figure** **1**A,B). We identified 855 genes differentially expressed in 3xTg‐AD mice compared to WT mice, highlighting significant transcriptome alternations associated with AD pathogenesis (Figure 1C). Notably, 44 genes (6 upregulated and 38 downregulated) were uniquely altered only in the X‐ray‐irradiated 3xTg‐AD mice, strongly correlating with the observed protective effects of LDIR (Figure 1D). Gene set enrichment analysis (GESA) revealed that X‐ray irradiation preferentially enriched signaling pathways involved in synapse assembly, regulation of synaptic plasticity, and long‐term synaptic potentiation (highlighted in red), while reducing activity in inflammatory responses associated with cytokine receptors, MHC class II protein complex, complement cascade, immune response, and cellular response to interferon‐gamma (highlighted in blue) (Figure 1E). GESA analysis indicated that X‐ray irradiation could mitigate proinflammatory responses and enhance synaptic function, thereby exerting a protective effect in the 3xTg‐AD mice.

We then assessed the clinical relevance of the LDIR‐induced protective effects by using the 44 differentially expressed genes (DEGs) as gene expression signatures to query the Library of Integrated Network‐Based Cellular Signatures database.^[^ ^24^, ^48^ ^]^ This analysis identified eight top‐ranked small molecules with connectivity and specificity scores above 90 (Figure S6A, Supporting Information). Based on literature research and commercial availability, we selected VUF6002 and prothionamide for testing their effects on cognitive function in sporadic AD mice. STZ mice treated with daily intraperitoneal (i.p.) injection of VUF6002 for 28 days showed significant improvement in cognitive performance (Figure S6B, Supporting Information). STZ mice treated with VUF6002 exhibited a notable increase in the spontaneous alternation rate in the Y‐Maze (Figure S6C, Supporting Information). During the Barnes maze, both sham controls and VUF6002‐treated STZ mice showed similar latencies to find the escape hole over the 4 days trial period (Figure S6D, Supporting Information). Furthermore, VUF6002‐treated STZ mice made fewer primary errors and reached the target hole faster than untreated STZ mice (Figure S6E–G, Supporting Information). By contrast, prothionamide did not show any beneficial effects on cognitive function recovery in the sporadic AD mice although prothionamide is known to cross the blood–brain barrier (BBB).^[^ ^49^, ^50^ ^]^ Pharmacokinetic analyses by liquid chromatography–tandem mass spectrometry (LC–MS/MS) revealed that VUF6002 rapidly crossed the BBB, detectable in the brain of adult mice 15 min after i.p. injection, with levels declining to undetectable after 8 h, indicating high BBB permeability (Figure S7A, Supporting Information). No published studies currently explore VUF6002's effects on the BBB, but our findings support its potential as a central nervous system (CNS)‐active agent.

We further tested the beneficial effects of VUF6002 in young familial AD mice (APP/PS1) by administrating daily i.p. injection starting at 7 months of age for 30 days (Figure S7B, Supporting Information). APP/PS1 mice expressed a chimeric mouse/human APP with the Swedish double mutations (K595N/M596L) and human presenilin 1 with the exon 9 deletion, developing Aβ deposits as early as 4 months of age and cognitive deficits by 6 months of age.^[^ ^51^, ^52^ ^]^ VUF6002 treatment completely reversed their cognitive deficits, as demonstrated in the Y‐Maze (Figure S7C, Supporting Information), NOR (Figure S7D, Supporting Information), and Morris water maze (MWM) (Figure S7E, Supporting Information). During the five days training in the MWM training, mice were trained to locate a hidden platform within an opaque water maze. Both WT and APP/PS1 + VUF6002 mouse groups showed progressive improvements, requiring less time to reach the platform, indicating comparable spatial learning abilities. In the probe test, where the platform was removed, the WT and APP/PS1 + VUF6002 mouse groups made significantly more crossings and spent more time in the target quadrant than the untreated APP/PS1 mice (Figure S7F–H, Supporting Information). Their swimming speeds were similar, suggesting that the increased crossings and time in the target quadrant reflect enhanced memory performance rather than increased locomotor activity (Figure S7I, Supporting Information).

LDIR treatment in young familial AD mice modulated gene expression in the hippocampus, upregulating 6 genes and downregulating 38 genes, as shown in Figure 1C,D. To validate these DEG changes in VUF6002‐treated young AD mice, quantitative polymerase chain reaction (qPCR) analysis of these 6 upregulated and top 20 downregulated DEGs (ranked by adjusted *p*‐value) was performed in the hippocampus of 8 months old APP/PS1 mice treated with VUF6002 for one month, during the same period as the behavior testing described above in Figure S7B (Supporting Information). VUF6002 modulated 19 of these DEGs, consistent with LDIR's transcriptomic gene signature (Figure S8A, Supporting Information). Furthermore, gene set overrepresentation analysis using DAVID Bioinformatics (NIH) revealed that VUF6002 significantly enriched pathways associated with synaptic function—such as regulation of presynaptic membrane potential (*Kcnj9*), synapse assembly (*Xlr3b*), and synaptic vesicle exocytosis *(Rims4)*—and suppressed immune response pathways *(Fmo1*, *H2‐ab1*, *B2m*, *Tnfrsf1a*, *H2‐K1*, *and Hmgcs2)*, including antigen processing and presentation via MHC class II (*H2‐ab1* and *B2m*) (Figure S8B, Supporting Information). These pathways closely align with LDIR's gene signature functional profile, confirming that VUF6002 effectively mimics LDIR's transcriptional and biological effects.

### 2.3 VUF6002 Reverses Cognitive Deficits and Major AD Pathogenesis in Two Aged Familial AD Mouse Lines

**Figure 2:**
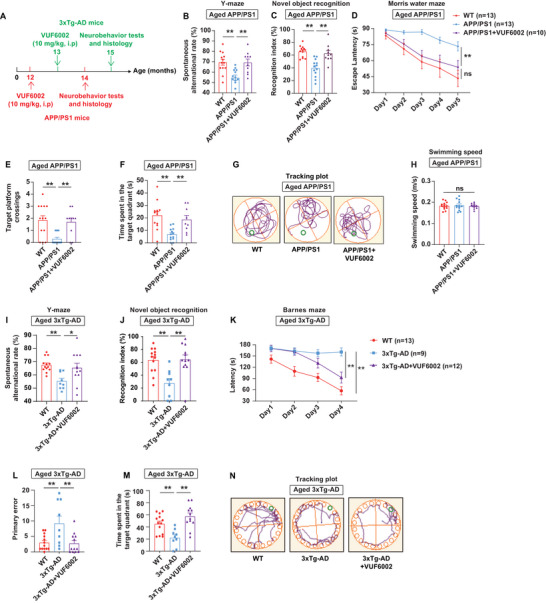
VUF6002 reverses cognitive deficits and major AD pathogenesis in two aged familial AD mouse lines. A) Aged APP/PS1 mice at 12 months and aged 3xTg‐AD mice at 13 months received VUF6002 (10 mg kg^−1^, intraperitoneal injection, i.p.) or 1% carboxyl methyl cellulose solution as vehicle control for two months. B) The Y‐maze test revealed a significant improvement in the percentage of spontaneous alternations in VUF6002‐treated aged APP/PS1 mice. C) VUF6002‐treated aged APP/PS1 mice showed novel object preference over a familiar one, similar to WT mice, indicating recovery of recognition memory. D) In the Morris water maze, aged APP/PS1 mice searched longer for the hidden platform, while VUF6002‐treated aged APP/PS1 mice behaved like WT mice during the 5 days training phase. E–G) In the day 6 probe trial, VUF6002‐treated aged APP/PS1 mice crossed the platform and spent time in the target quadrant more frequently than aged APP/PS1 mice, comparable to WT mice. H) Swim speed was similar among all experimental groups. I) VUF6002‐treated aged 3xTg‐AD mice showed significantly greater improvement in the Y‐maze test compared to untreated mice, indicating a reversal of working memory deficits. J) VUF6002‐treated aged 3xTg‐AD mice showed no difference in novel object exploration when compared to WT mice. K–N) In the Barnes maze, aged 3xTg‐AD mice exhibited significant spatial learning and memory deficits compared to WT mice, evidenced by longer escape latencies, higher primary error rates, and less time in the target quadrant. VUF6002 treatment completely reversed these cognitive deficits. The green highlight circles indicate the locations of the hidden platform or escape box in (G) and (N). Mean ± SEM (*n* = 9–13 per group). * *p* < 0.05; ** *p* < 0.01; ns: not significant; one‐way ANOVA followed by Tukey post hoc test (B, C, E, F, H–J, L, and M); two‐way repeated‐measures ANOVA followed by Tukey post hoc test (D and K).

Aging is the primary risk factor for AD, with emerging evidence supporting the age‐dependent amyloid cascade hypothesis, which suggests that amyloid pathology precedes tauopathy.^[^ ^53^ ^]^ To evaluate the translational potential of VUF6002 in advanced AD, we treated aged APP/PS1 (12 months old) and 3xTg‐AD (13 months old) mice with VUF6002 for 2 months (**Figure** **2**A). Consistent with earlier results in 8 months old APP/PS1 mice, aged APP/PS1 mice receiving VUF6002 exhibited behavior comparable to WT mice. In the Y‐maze test, VUF6002 fully restored cognitive function and exploratory behavior, with treated aged APP/PS1 mice showing a significantly higher spontaneous alternation rate than untreated ones (Figure 2B). Similarly, in the NOR test, VUF6002‐treated aged APP/PS1 mice spent more time exploring a new object than a familiar one, mirroring WT behavior and indicating a complete recognition memory recovery (Figure 2C). In the MWM, aged APP/PS1 mice took longer to find the hidden platform during training, whereas VUF6002‐treated aged APP/PS1 mice performed similarly to WT mice (Figure 2D). During day 6 probe trial, untreated aged APP/PS1 mice crossed the platform location less frequently and spent less time in the target quadrant compared to WT controls. By contrast, VUF6002‐treated mice showed performance compared to WT mice (Figure 2E–G). Swim speeds across all groups were similar, indicating motor functions were unaffected (Figure 2H).

The 3xTg‐AD mice is widely used for studying AD, as it carries mutations in the *APP*, *MAPT*, and *PSEN1* genes, allowing it to develop both Aβ plaques and neurofibrillary tangles.^[^ ^42^ ^]^ Strikingly, VUF6002‐treated aged 3xTg‐AD mice showed substantially higher alterations in the Y‐maze test compared to aged 3xTg‐AD mice, indicating that VUF6002 treatment effectively reversed working memory deficits in these aged 3xTg‐AD mice (Figure 2I). Consistent with this, aged 3xTg‐AD mice normally spend less time exploring the novel object compared to the familiar one; however, VUF6002‐treated aged 3xTg‐AD mice spent more time exploring the novel object, with no significant difference in exploration time between VUF6002‐treated aged 3xTg‐AD mice and WT controls (Figure 2J). In the Barnes maze, untreated aged 3xTg‐AD mice exhibited marked deficits in spatial learning and memory, evidenced by longer escape latencies during training, higher primary error rate, and reduced time in the target quadrant during the probe trial. Treatment with VUF6002 fully reversed these cognitive deficits in aged 3xTg‐AD mice, restoring performance to levels comparable to WT mice (Figure 2K–N).

**Figure 3:**
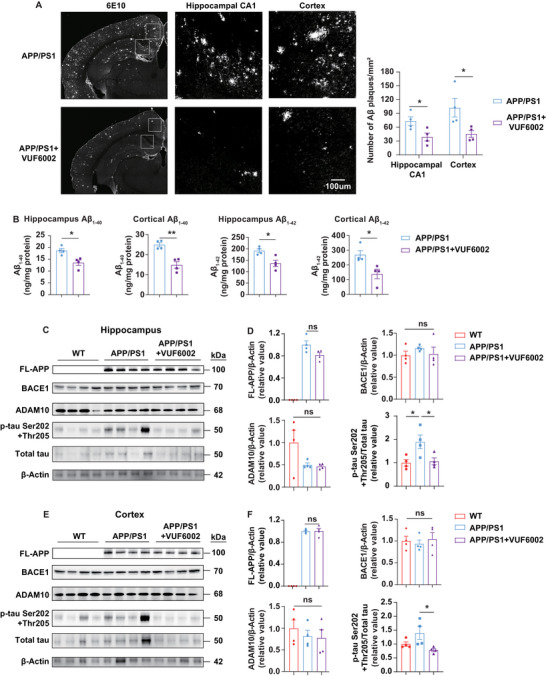
VUF6002 reduces amyloid pathology in aged APP/PS1 mice without altering APP metabolism. A) Representative confocal photomicrographs showing the clearance of 6E10‐stained Aβ plaques from the hippocampus CA1 area and cortex of 14 months old APP/PS1 after two months of daily VUF6002 treatment. The numbers of Aβ plaques mm^2^ was quantified using NIS‐Elements software (Nikon). Scale bar: 100 µm. B) The concentrations of soluble human Aβ_1–40_ and Aβ_1–42_ in hippocampal and cortical brain lysates were quantitatively measured by an Aβ ELISA assay after two months of daily VUF6002 treatment. C–F) Representative Western blot images showed full‐length (FL)‐APP, BACE1, ADAM10, phosphorylated tau at Ser202 and Thr205 (p‐tau Ser202 + Thr205) and Total tau in the hippocampus (C, D) and the cortex (E, F) of 14 months old WT mice, vehicle‐treated APP/PS1 mice, and VUF6002‐treated APP/PS1 mice. Two months of VUF6002 treatment did not affect the amyloidogenic pathway but significantly reduced p‐tau Ser202 + Thr205 levels in the hippocampus and cortex of aged APP/PS1 mice. Each lane represents a brain sample collected from one mouse. FL‐APP/BACE1/ADAM10 expression was normalized to β‐Actin, p‐tau Ser202 + Thr205 expression was normalized to Total tau. Mean ± SEM (*n* = 4 per group). * *p* < 0.05; ** *p* < 0.01; ns: not significant; student's *t*‐test (A,B); one‐way ANOVA followed by Tukey post hoc test (D and F).

To explore the putative link between behavioral improvement and age‐related Aβ plaques and tauopathy, we performed a detailed analysis of Aβ deposition and metabolism, as well as p‐tau, in two advanced stage AD mouse models. Immunohistochemistry analysis demonstrated that VUF6002 treatment significantly reduced Aβ plaque accumulation, indicated by decreases in the numbers of 6E10‐labeled Aβ plaques in the hippocampus (47%) and cortex (56%) of aged APP/PS1 mice (14 months old) after two months of treatment (**Figure** **3**A). These histological findings were further validated by Aβ ELISA assays, which showed reductions in soluble Aβ_1–40_ and Aβ_1–42_ levels in both the hippocampus and cortex of aged APP/PS1 mice (Figure 3B). Aβ is produced through the sequential cleavage of APP by beta‐secretase and gamma‐secretase, a process known as APP metabolism.^[^ ^54^ ^]^ Western blot analysis of key proteins involved in this metabolomic pathway—including full‐length APP (FL‐APP), β‐site APP cleaving enzyme (BACE, also known as beta‐secretase), and ADAM10 (an alpha‐secretase that processes APP protein via a nonamyloidogenic pathway^[^ ^55^ ^]^revealed no significant changes in their expression levels, indicating that APP metabolism was unaffected by VUF6002 treatment. Since tau hyperphosphorylation occurs in response to Aβ accumulation during AD progression,^[^ ^56^ ^]^ we assessed tau phosphorylation at Ser202 and Thr205. Remarkably, the phosphorylation levels in the hippocampus (Figure 3C,D) and cortex (Figure 3E,F) of VUF6002‐treated aged APP/PS1 mice were completely abolished.

Furthermore, similar beneficial effects were observed in a second AD mouse model, the 15 months old 3xTg‐AD mice, including increased Aβ plaque clearance (Figure S9A, Supporting Information), decreased soluble Aβ_1–40_ and Aβ_1–42_ levels in the hippocampus and cortex (Figure S9B,C, Supporting Information), and reduced tau phosphorylation, as confirmed by immunohistochemistry (Figure S9D, Supporting Information) and Western blot analyses (Figure S9E,F, Supporting Information). Collectively, these results support the conclusion that VUF6002 effectively ameliorates major Aβ pathology without affecting APP metabolism.

### 2.4 VUF6002 Promotes Microglial Aβ and p‐Tau Phagocytosis

**Figure 4:**
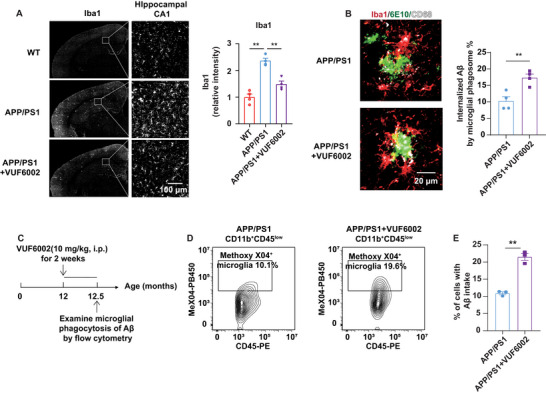
VUF6002 inhibits microgliosis and promotes microglial phagocytosis of Aβ and phosphorylated tau in aged APP/PS1 mice. A) Representative confocal photomicrographs showed a reduction in microgliosis, as indicated by Iba1 immunoreactivity in the hippocampus CA1 area of 14 months old APP/PS1 mice after two months of VUF6002 treatment. The relative intensity of Iba1 was measured using NIS‐Elements software (Nikon). Scale bar: 100 µm (*n* = 4 per group). B) 3D surface reconstruction colocalization analysis of Iba1‐positive microglia (red), 6E10‐positive Aβ plaques (green) and CD68‐positive microglial phagosomes (white) demonstrated enhanced Aβ engulfment in the hippocampal CA1 region of VUF6002‐treated APP/PS1 mice. The colocalized volume of microglial lysosomes (Iba1^+^CD68^+^) with Aβ plaques was normalized to the volume of Aβ plaques in vehicle‐treated APP/PS1 mice (*n* = 64 Aβ plaques) and VUF6002‐treated APP/PS1 mice (*n* = 80 Aβ plaques). Scale bar: 20 µm (*n* = 4 per group). C) Aged APP/PS1 mice at 12 months were treated with VUF6002 for 2 weeks. Microglial phagocytosis of Aβ was validated using a flow cytometry‐based approach to quantify the Aβ phagocytic capacity of microglia in vivo. D,E) The scatterplot showed the proportion of the CD11b^+^CD45^low^ resident microglia containing Methoxy‐X04‐labeled Aβ fibrils [methoxy‐X04^+^ (MeX04^+^)] in 12.5 months old vehicle‐treated or VUF6002‐treated APP/PS1 mice (*n* = 6 per group). Cortex and hippocampus from two mice were pooled for each flow cytometry experiment, and three independent experiments were performed. Mean ± SEM. * *p* < 0.05; ** *p* < 0.01; one‐way ANOVA followed by Tukey post hoc test (A); student *t*‐test (B and E).

During AD progression, microglia are essential for phagocytosing Aβ aggregates, which helps to reduce Aβ levels and limit plaque formation. Interestingly, VUF6002 treatment in aged AD mice significantly reduced Aβ accumulation without affecting its metabolism or production. To understand how VUF6002 influences microglial activity, we examined microglial activity and function two months posttreatment. In aged APP/PS1 mice, the number of Iba1‐positive activated microglia was markedly increased in the hippocampal CA1 region compared to control mice, indicating microgliosis. However, VUF6002 reduced microgliosis to levels comparable to control mice (**Figure** **4**A). Since microglia are the primary cells responsible for clearing extracellular Aβ, we performed 3D surface reconstruction colocalization analysis of Iba1‐positive microglia and 6E10‐positive Aβ plaques in 14 months old APP/PS1 mice.^[^ ^57^, ^58^ ^]^ Results showed significantly enhanced Aβ engulfment, indicated by overlapping Iba1 and 6E10 signals, in the hippocampal CA1 region of VUF6002‐treated mice. To confirm if this increased microglial activity facilitated Aβ internalization, we quantified the colocalization of Aβ within CD68‐positive microglial phagosomes. The immunoreactivity of CD68‐positive microglial phagosomes strongly overlapped with 6E10‐positive Aβ plaques within Iba1‐positive microglia of in VUF6002‐treated mice, compared to vehicle‐treated controls (Figure 4B).

To validate these findings, all experiments were repeated in a second AD mouse model, the 15 months old 3xTg‐AD mice. VUF6002 again demonstrated strong beneficial effects by reducing microgliosis (Figure S10A, Supporting Information) and enhancing microglia‐mediated Aβ clearance (Figure S10B, Supporting Information). Additionally, emerging evidence indicates that microglial phagocytosis also plays a key role in clearing extracellular p‐tau, thereby preventing its spread and slowing AD progression.^[^ ^59^, ^60^, ^61^, ^62^ ^]^ Our 3D surface reconstruction studies further revealed that Iba1^+^CD68^+^ microglial phagosomes not only surrounded but also highly colocalized with AT8‐positive p‐tau (Ser202 and Thr205) (Figure S10C, Supporting Information).

To examine the contribution and specificity of resident microglia in Aβ clearance following VUF6002 treatment, we used flow cytometry to quantify microglial Aβ phagocytic capacity in vivo two weeks posttreatment (Figure 4C). We intraperitoneally injected 12.5 months old aged APP/PS1 mice with methoxy‐X04 and analyzed the subset of CD11b^+^ myeloid cells with low CD45 expression (CD11b^+^CD45^low^) (Figure 4D), which allowed us to distinguish resident microglia from other myeloid cell populations.^[^ ^63^, ^64^ ^]^ We then evaluated the number of microglia that had internalized Aβ plaques by measuring the uptake of methoxy‐X04‐labeled Aβ. Our results revealed a significant increase—doubling the methoxy‐X04 Aβ signal—in microglia of VUF6002‐treated aged APP/PS1 mice compared to vehicle‐treated controls, indicating that VUF6002 enhanced microglial Aβ phagocytosis in vivo (Figure 4E).

### 2.5 Microglial Deletion of *Hrh4*, but Not Neuronal Deletion of *Hrh4*, Rescues Cognitive Function Deficits in AD Mice

In AD patients, elevated histamine levels have been detected in cerebrospinal fluid and multiple brain regions, including the hippocampus, frontal cortex, temporal cortex, and basal ganglia.^[^ ^65^, ^66^ ^]^ A recent study reported that intracerebroventricular injection of histamine in rats activated microglia and increased proinflammatory cytokines.^[^ ^67^ ^]^ In the current study, we investigated the effects of antagonizing the histamine receptor H_4_R via systemic administration of VUF6002 on neurons and glial cells. To explore the cell type‐specific effects of *Hrh4‐*deletion in AD progression, we evaluated the beneficial effects of neuronal and microglial *Hrh4*‐deletion in ameliorating cognitive deficits and pathogenesis. We and others have demonstrated the superior ability of adeno‐associated virus serotype 2/9 (AAV2/9) to transduce neurons in various tissues: in the brain following intracerebroventricular injection,^[^ ^68^, ^69^ ^]^ in the dorsal root ganglion after sciatic nerve injection,^[^ ^24^, ^70^, ^71^ ^]^ and in the retina via intravitreal injection.^[^ ^70^, ^72^ ^]^ To knock down *Hrh4* expression in the brain, we delivered a recombinant AAV2/9 vector carrying shRNA targeting *Hrh4*. To validate the specificity of the knockdown, a scrambled shRNA vector was used as a control (AAV2/9–scr‐shRNA). Both vectors were microinjected into the cortex and hippocampus (CA1 and CA3 regions) of 6 months old APP/PS1 mice. Two months post‐injection, we assessed the neuronal knockdown of *Hrh4* in NeuN‐positive neurons of APP/PS1 mice via flow cytometry (Figure S11A, Supporting Information). NeuN‐positive neuronal nuclei were isolated from the cortex and hippocampi using FACS (Figure S11B, Supporting Information). Importantly, our analysis revealed a significant 74% reduction of *Hrh4* expression in FACS‐sorted NeuN‐positive neurons from mice treated with AAV2/9–*Hrh4*–shRNA, compared to those NeuN‐positive neurons in the AAV2/9–scr‐shRNA‐treated APP/PS1 mice (Figure S11C, Supporting Information). To investigate the effect of neuronal *Hrh4* deletion on cognitive functions, hippocampal‐dependent learning and memory were assessed using the MWM in APP/PS1 mice following neuronal *Hrh4* knockdown in the cortex and hippocampus. During the training, both AAV2/9–*Hrh4*–shRNA‐treated and AAV2/9–scr‐shRNA‐treated APP/PS1 mice demonstrated similar learning abilities (Figure S11D, Supporting Information). At 8 months of age, the performance of the AAV2/9–*Hrh4*–shRNA‐treated APP/PS1 mice showed no significant difference from the control group across all measures, including platform crossings (Figure S11E, Supporting Information), time spent in the target quadrant (Figure S11F,G, Supporting Information), and swimming speed (Figure S11H, Supporting Information). As expected, analysis of Aβ deposition revealed no difference between the AAV2/9–*Hrh4*–shRNA‐treated mice and AAV2/9–scr‐shRNA‐treated APP/PS1 mice (Figure S11I, Supporting Information). These results indicate that neuronal *Hrh4* knockdown does not improve hippocampal‐dependent spatial memory in AD mice.

To further investigate the role of microglial H_4_R in AD, we silenced *Hrh4* expression specifically in microglia within the cortex and hippocampus using a lentiviral vector encoding *Hrh4*‐targeted shRNA (Lenti–*Hrh4*–shRNA) under the control of a fractalkine receptor gene promoter Cx3cr1, which selectively labels microglia.^[^ ^73^, ^74^, ^75^ ^]^ Similarly, Lenti–*Hrh4*–shRNA or Lenti–scr‐shRNA was injected into the cortex and hippocampus regions (CA1 and CA3) of 6 months old APP/PS1 mice (Figure S12A, Supporting Information). FACS‐sorted CD11b^+^CD45^low^ resident microglia (Figure S12B, Supporting Information) confirmed high H_4_R expression in the Lenti–scr‐shRNA‐treated mice, while H_4_R protein levels were effectively knocked down in microglia from the Lenti–*Hrh4*–shRNA‐treated APP/PS1 mice (Figure S12C, Supporting Information). To confirm that the microglial deletion of *Hrh4* was specific and did not affect neuronal expression of H_4_R, we isolated NeuN‐positive neurons from the same mice via FACS for further analysis (Figure S12D, Supporting Information). The neuronal H_4_R expression remained unchanged in mice treated with Lenti–*Hrh4*–shRNA compared to those treated with Lenti–scr‐shRNA (Figure S12E, Supporting Information). Following this, mice were subjected to the MWM to evaluate the effect of microglial *Hrh4* deletion on spatial learning and memory. During the training, both Lenti–*Hrh4*–shRNA‐treated APP/PS1 and WT mice showed a significant reduction in the time required to reach the platform (Figure S12F, Supporting Information). In the probe trial, only the Lenti–*Hrh4*–shRNA‐treated APP/PS1 mice exhibited a significant increase in platform crossing (Figure S12G, Supporting Information) and greater time spent in the target quadrant (Figure S12H,I, Supporting Information), compared to the Lenti–scr‐shRNA‐treated APP/PS1 control mice. Importantly, all mouse groups exhibited similar motor functions, as evidenced by the absence of significant differences in swimming speed across the different mouse groups (Figure S12J, Supporting Information).

### 2.6 Microglial *Hrh4* Deletion Reverses Cognitive Function Deficits and Major AD Pathogenesis in Aged AD Mice through the Activation of TGF‐β1

**Figure 5:**
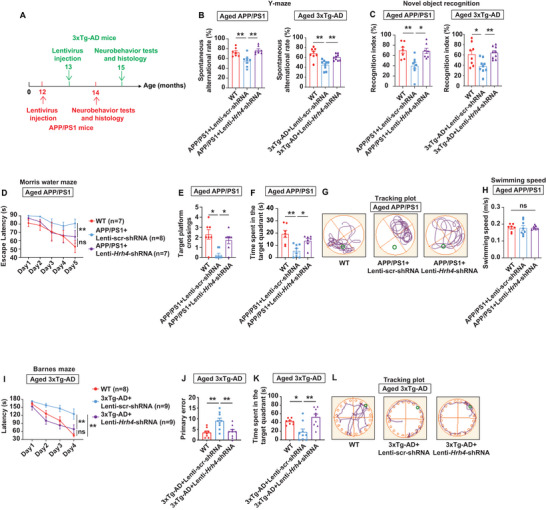
Microglial deletion of *Hrh4* rescues cognitive deficits of 14 months old APP/PS1 mice and 15 months old 3xTg‐AD mice. A) APP/PS1 mice at 12 months and 3xTg‐AD mice at 13 months were intracranially injected with lentivirus to knockdown *Hrh4* in microglia in the cortex and hippocampus (CA1 and CA3). Two months post‐injection, neurobehavior tests were performed to assess cognitive function recovery. B) Aged APP/PS1 and 3xTg‐AD mice showed significant learning and memory impairments in the Y‐maze. By contrast, microglial deletion of *Hrh4* in these mice resulted in spontaneous arm entry alternation similar to their age‐matched controls. C) Microglial deletion of *Hrh4* reversed cognitive deficits in the novel object recognition test, restoring the recognition index to control levels in both AD mouse models. D–H) In APP/PS1 mice, microglial deletion of *Hrh4* also improved spatial memory in the Morris water maze, as evidenced by shorter escape latencies (D), an increased number of platform crossings (E), and more time spent in the target quadrant (F, G), with swimming speed remaining unchanged across all mouse groups (*n* = 7–8 per group). I–L) Assessment of spatial memory in the Barnes maze revealed no significant differences between wildtype and Lenti–*Hrh4*–shRNA‐treated 3xTg‐AD mice in terms of escape latency during the 4 days training sessions (I), primary errors (J), or time spent in the target quadrant during the subsequent probe trial (K, L) (*n* = 8–9 per group). Mean ± SEM. * *p* < 0.05; ** *p* < 0.01; ns: not significant; two‐way repeated‐measures ANOVA followed by Tukey post hoc test (D and I); one‐way ANOVA followed by Tukey post hoc test (B, C, E, F, H, J, K). scr‐shRNA: scrambled shRNA.

Given the promising effects of microglial *Hrh4* deletion in early stages of AD, it is critical to further evaluate its potential to reverse cognitive impairments in the advanced stage of AD, which are highly relevance to AD patients. To this end, we applied the same treatment paradigm used for 6 months old APP/PS1 mice to two aged AD mouse models. Starting at 12 months of age for APP/PS1 and 13 months for 3xTg‐AD mice, we treated the aged animals with Lenti–*Hrh4*–shRNA for 2 months (**Figure** **5**A). Aged APP/PS1 and 3xTg‐AD mice showed significant impairments in learning and memory in the Y‐maze. By contrast, microglial deletion of *Hrh4* in aged APP/PS1 and 3xTg‐AD mice led to a significant improvement in spontaneous alternation behavior, comparable to that of their aged‐matched control mice (Figure 5B). In the NOR test, both aged APP/PS1 and 3xTg‐AD mice failed to distinguish between novel and familiar objects. Notably, However, deletion of *Hrh4* in microglia significantly rescued this cognitive deficit, increasing the recognition index to baseline levels seen in controls (Figure 5C). In APP/PS1 mice, microglial *Hrh4* deletion also reversed spatial memory impairments in the MWM, evidenced by reduced escape latency (Figure 5D), increased platform crossings (Figure 5E), and longer time spent in the target quadrant during probe trials (Figure 5F,G). Swimming speed remained unaffected (Figure 5H). The Barnes maze further confirmed these findings, showing no significant differences between WT and Lenti–*Hrh4*–shRNA‐treated 3xTg‐AD mice in escape latency across training days, as well as the number of primary errors and time spent in the target quadrant during the probe trial (Figure 5I–L).

**Figure 6:**
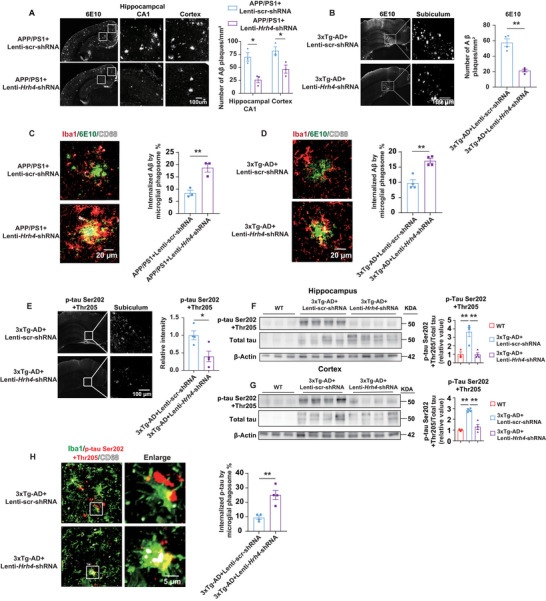
Microglial deletion of *Hrh4* increases microglial phagocytosis of Aβ and phosphorylated tau. A,B) Deletion of *Hrh4* significantly decreased Aβ burden, as indicated by a marked reduction in 6E10 immunostaining in the hippocampus and cortex of 14 months old APP/PS1 mice two months after lentivirus injection (A) (*n* = 3 per group), as well as in the subiculum of aged 3xTg‐AD mice (B) (*n* = 4 per group). Scale bar: 100 µm. C,D) 3D reconstruction imaging demonstrated that microglial deletion of *Hrh4* led to a substantial increase in the internalization of 6E10‐positive Aβ plaques (green) by CD68‐positive microglial phagosomes (white) in Iba1‐positive (red) microglia in the hippocampal CA1 area of aged APP/PS1 mice (C) (*n* = 3 per group) and subiculum area of aged 3xTg‐AD mice (D) (*n* = 4 per group). The volume of colocalized microglial lysosomes (Iba1^+^CD68^+^) with Aβ plaques was normalized to the Aβ plaque volume (*n* = 125 Aβ plaques from APP/PS1 + Lenti–scr‐shRNA; *n* = 104 Aβ plaques from APP/PS1 + Lenti–*Hrh4*–shRNA) in (C) (*n* = 64 Aβ plaques from 3xTg‐AD + Lenti–scr‐shRNA; *n* = 64 Aβ plaques from 3xTg‐AD + Lenti–*Hrh4*–shRNA) in (D). Scale bar: 20 µm. E) Representative confocal images of anti‐AT8‐positive phosphorylated tau at Ser202 and Thr205 from 3xTg‐AD mice, treated with Lenti–scr‐shRNA (*n* = 4) or Lenti–*Hrh4*–shRNA (*n* = 4 per group), demonstrated a reduction in p‐tau levels. Scale bar: 100 µm. F,G) Western blot analysis of phosphorylated tau levels at Ser202 and Thr205 in the (F) hippocampus and (G) cortex across different treatment groups confirmed the immunohistochemistry results. The p‐tau expression was normalized to Total tau. Each lane represents a brain sample collected from one mouse (*n* = 4 per group). H) 3D reconstruction imaging revealed a substantial increase in microglial phagocytosis of p‐tau in the subiculum of aged 3xTg‐AD mice. This imaging showed microglia (Iba1, green), lysosomes (CD68, white), and p‐tau Ser202 + Thr205 (AT8, red). The colocalized volume of microglial lysosomes (Iba1^+^CD68^+^) and p‐tau were normalized to the volume of p‐tau in aged 3xTg‐AD mice (*n* = 64 images) and in 3xTg‐AD mice with microglial *Hrh4* deletion (*n* = 64 images) (*n* = 4 per group). Mean ± SEM. * *p* < 0.05; ** *p* < 0.01; student's *t*‐test in (A–E and H); one‐way ANOVA followed by Tukey post hoc test in (F and G). scr‐shRNA: scrambled shRNA.

To determine whether the cognitive improvement observed after microglial *Hrh4* deletion were associated with reductions in Aβ and tau pathology, we quantified Aβ plaques in the brains of Lenti–*Hrh4*–shRNA‐treated aged APP/PS1 and 3xTg‐AD mice. Microglial *Hrh4* deletion significantly reduced Aβ burden, as indicated by a marked reduction in 6E10 immunostaining in the hippocampus and cortex of aged APP/PS1 mice (**Figure** **6**A), as well as in the subiculum of aged 3xTg‐AD mice (Figure 6B). More strikingly, representative 3D reconstruction images revealed that microglial *Hrh4* deletion markedly enhanced the internalization of 6E10‐positive Aβ plaques by CD68‐positive microglial phagosome within Iba1‐positive microglia, increasing this by 123% in aged APP/PS1 mice (Figure 6C), and by 75% in aged 3xTg‐AD mice (Figure 6D). Of note, *Hrh4* deletion significantly reduced p‐tau deposition in the subiculum of aged 3xTg‐AD mice by 60%, as shown by immunohistochemistry analysis (Figure 6E). These findings were confirmed by Western blot analysis, which revealed a substantial decrease in p‐tau levels in both the hippocampus (Figure 6F) and cortex (Figure 6G). To assess microglial phagocytic activity, we quantified the colocalization of AT8‐postive p‐tau within Iba1^+^CD68^+^ microglial phagosomes in the subiculum of *Hrh4*‐deleted 3xTg‐AD mice. The percentage of colocalization was significantly increased in the *Hrh4*‐deleted 3xTg‐AD mice (Figure 6H).

**Figure 7:**
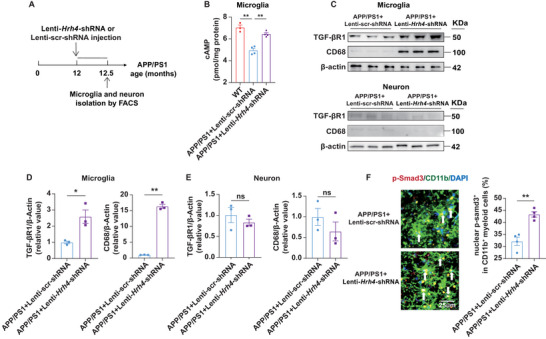
Microglia, but not neuronal deletion of *Hrh4*, activates the cAMP/TGF‐β1/Smad3 signaling pathway. A) We performed fluorescence‐activated cell sorting (FACS) to isolate microglia from 12.5 months old APP/PS1 mice that received intracranial injections of either Lenti–*Hrh4*–shRNA or Lenti–scr‐shRNA into the cortex and hippocampus (CA1 and CA3 regions) for two weeks. B) The deletion of *Hrh4* in microglia resulted in a restoration of cAMP levels to those measured in wild‐type mice. Cortex and hippocampus from two mice were pooled for each flow cytometry experiment and cAMP measurement (*n* = 6‐8 per group). C) Western blot analysis was performed to assess the protein expression of TGF‐β receptor 1 (TGF‐βR1) and the phagocytic marker CD68 in FACS‐sorted microglia and FACS‐sorted neurons (*n* = 6 per group). Each lane represents a sample collected the cortex and hippocampus, processed by FACS from two mice. D) TGF‐βR1 and CD68 were significantly upregulated in the microglia of aged Lenti–*Hrh4*–shRNA‐treated APP/PS1 mice, whereas a low CD68 expression was observed in Lenti–scr‐shRNA‐treated APP/PS1 mice. E) TGF‐βR1 was expressed at very low levels and CD68 was nearly undetectable in the neurons of all mouse groups. TGF‐βR1 or CD68 expression were normalized to β‐Actin. F) *Hrh4* deletion increased the percentage of myeloid cells with p‐Smad3 translocated into the nuclei in aged APP/PS1 mice, as shown by immunostaining of p‐Smad3 (red), CD11b (green), and DAPI (blue) (*n* = 4 per group). Mean ± SEM. * *p* < 0.05; ** *p* < 0.01; ns: not significant; one‐way ANOVA followed by Tukey post hoc test (B). Student's *t*‐test (D–F). scr‐shRNA: scrambled shRNA.

The H_4_R is a G protein‐coupled receptor complex composed of α‐ and β‐ and γ‐subunits.^[^ ^76^ ^]^ It primarily signals through G_αi/o_ proteins, with H_4_R activation reducing cAMP levels through G_αi/o_ coupling. This reduction in cAMP is crucial for maintaining synaptic plasticity, learning, and memory. Enhancing the cAMP signaling pathway or inhibiting cAMP degradation can reverse spatial memory deficits.^[^ ^77^, ^78^ ^]^ Interference with the classical TGF‐β signal transduction pathway may serve as a potential mechanism, as intracellular cAMP levels are known to play a significant role in the TGF‐β1/Smad3 signaling pathway.^[^ ^79^, ^80^ ^]^ Based on these findings, we hypothesize that *Hrh4* deletion in microglia could increase cAMP levels, which in turn enhances microglial phagocytosis via activation of the TGF‐β1/Smad3 pathway. To test this, we used FACS to isolate microglia from 12.5 months old APP/PS1 mice injected with either Lenti–*Hrh4*–shRNA or Lenti–scr‐shRNA into the cortex and hippocampus (CA1 and CA3 regions) (**Figure** **7**A). We first measured microglial cAMP levels using a mouse cAMP detection ELISA kit. Compared to microglia sorted from age‐matched WT mice, the cAMP levels in microglia from aged APP/PS1 mice was significantly lower. Notably, deletion of *Hrh4* in microglia restored their cAMP levels to those observed in WT mice (Figure 7B). We then performed Western blot analysis to examine TGF‐βR1 and the phagocytic marker CD68 protein expression in FACS‐isolated microglia and neurons (Figure 7C). CD68 is a transmembrane glycoprotein functioning as a microglial scavenger receptor for Aβ clearance.^[^ ^57^, ^81^ ^]^ Strikingly, both TGF‐βR1 and CD68 were substantially upregulated in the microglia of Lenti–*Hrh4*–shRNA‐treated aged APP/PS1 mice, whereas nearly no CD68 expression was detected in the microglia of Lenti–scr‐shRNA‐treated aged APP/PS1 mice (Figure 7D). As expected, TGF‐βR1 was expressed at very low levels, and CD68 was almost undetectable in neurons, with no significant differences observed between the lentivirus treatment groups (scr or *Hrh4*), confirming the specificity of microglial deletion of *Hrh4* (Figure 7E). Smad3, a downstream effector of TGF‐βR1, plays a central role in the TGF‐β signaling pathway. Upon binding of TGF‐β to its transmembrane receptors, Smad3 is phosphorylated (p‐Smad3), which drives its translocation into the nucleus, where it binds DNA to regulate target gene expression.^[^ ^82^ ^]^ Immunohistochemistry revealed that microglial deletion of *Hrh4* significantly increased the percentage of myeloid cells (CD11b^+^DAPI^+^) colocalizing with p‐Smad3 in the hippocampal CA1 region. This indicates that *Hrh4* deletion promoted the translocation of p‐Smad3 into the nucleus of myeloid cells in aged APP/PS1 mice (Figure 7F). Supporting this, LDIR treatment also upregulated TGF‐βR1 expression in aged 3xTg‐AD mice (Figure S13, Supporting Information), suggesting mechanistic consistency between LDIR and *Hrh4* deletion in mediating therapeutic effects against AD.

**Figure 8:**
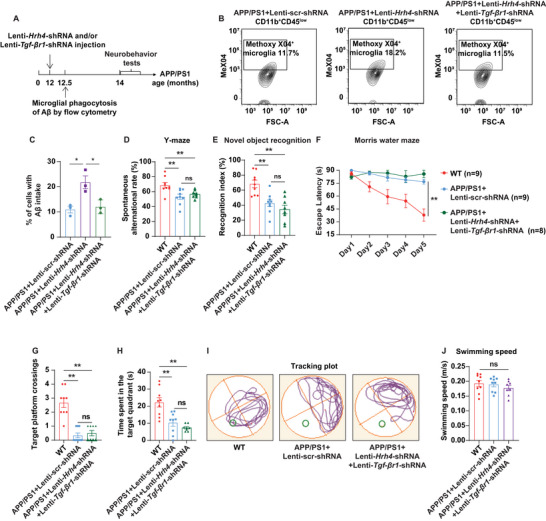
Deletion of *Tgf‐βr1* in microglia impairs both phagocytosis and cognitive functional recovery in Lenti–*Hrh4*–shRNA‐treated APP/PS1 mice. A) At 12 months of age, APP/PS1 mice received intracranial injections of lentivirus to knockdown microglial *Hrh4* and/or *Tgf‐βr1* in the cortex and hippocampus (CA1 and CA3 regions). Two weeks after the lentivirus injections, in vivo microglial phagocytosis of Aβ was quantified using flow cytometry, and neurobehavioral tests were performed to evaluate cognitive function two months post lentivirus injection. B,C) Microglial deletion of *Hrh4* significantly increased the uptake of methoxy‐X04‐labeled Aβ by resident microglia (CD11b^+^CD45^low^), an effect that was completely abolished by the additional deletion of *Tgf‐βr1* in Lenti–*Hrh4*–shRNA‐treated APP/PS1 mice (*n* = 6 per group). Cortex and hippocampus from two mice were pooled for each flow cytometry experiment, and three independent experiments were performed. D–J) There were no significant differences between the Lenti–scr‐shRNA control group and the Lenti–*Hrh4*–shRNA + Lenti–*Tgf‐βr1*–shRNA treatment group in APP/PS1 mice. The deletion of *Tgf‐βr1* in Lenti–*Hrh4*–shRNA‐treated APP/PS1 mice completely eliminated the beneficial effects of *Hrh4* deletion in the Y‐maze (D), novel object recognition (E), and Morris water maze (F–I) tests, with no effect on swimming speed across all mouse groups (J) (*n* = 8–9 per group). Mean ± SEM, * *p* < 0.05, ** *p* < 0.01; ns: not significant; one‐way ANOVA followed by Tukey post hoc test (C–E, G, H, J). Two‐way repeated‐measures ANOVA followed by Tukey post hoc test (F). WT: wildtype mice.

To further explore the role of the TGF‐β signaling pathway in mediating the beneficial effects of microglial *Hrh4* deletion in AD mice, we quantified microglial Aβ phagocytic capacity in vivo following injection of Lenti–scr‐shRNA, Lenti–*Hrh4*–shRNA, and/or Lenti–*Tgf‐βr1*–shRNA into the cortex and hippocampus (CA1 and CA3 regions) of aged APP/PS1 mice (**Figure** **8**A). Western blot analysis confirmed effective knockdown of TGF‐βR1, with a 62% reduction in FACS‐sorted microglia from aged APP/PS1 mice (Figure S14, Supporting Information). Microglial deletion of *Hrh4* significantly increased the uptake of methoxy‐X04‐labeled Aβ by resident microglia (CD11b^+^CD45^low^), but this promoting effect was completely abolished when *Tgf‐βr1* was also knocked down in microglia of Lenti–*Hrh4*–shRNA‐treated APP/PS1 mice (Figure 8B,C). Next, we assessed whether modulation of microglial activity and Aβ clearance through TGF‐β signaling translated into improved cognitive function in aged APP/PS1 mice. Strikingly, behavioral tests demonstrated that there were no significant differences between the APP/PS1 mice treated with Lenti–scr‐shRNA and those APP/PS1 mice with combined *Hrh4* and *Tgf‐βr1* knockdown (Lenti–*Hrh4*–shRNA + Lenti–*Tgf‐βr1*–shRNA). The beneficial effects of *Hrh4* deletion on spatial memory and recognition were completely abolished by *Tgf‐βr1* knockdown, as evidenced by the Y‐maze (Figure 8D), NOR (Figure 8E), and MWM tests (Figure 8F–I), without affecting swimming speed (Figure 8J). These findings provide compelling evidence that the TGF‐β signaling pathway is directly involved in mediating the therapeutic effects of microglial *Hrh4* deletion in AD.

Finally, considering the implicated role of TGF‐β signaling abnormalities in AD pathogenesis, we examined TGF‐βR1 expression in human brain tissue datasets. In the GSE5281 dataset, which included 16 AD patients and 12 healthy controls, human *Tgf‐βr1* expression was significantly downregulated by 37% in the middle temporal gyrus—a region associated with recognition processing in humans^[^ ^83^, ^84^ ^]^ (Figure S15A, Supporting Information). Similarly, analysis of the GSE15222 dataset (177 AD patients and 187 controls) revealed a 17% transcriptional down‐regulation of cortical *Tgf‐βr1* expression in AD patients compared to healthy individuals (Figure S15B, Supporting Information). These data suggest that impairment of TGF‐β1 signaling may contribute to cognitive deficits observed in AD.

## 3 Discussion

Histamine is a biogenic amine that acts as a potent inflammatory mediator in both peripheral allergic responses and CNS inflammation.^[^ ^85^, ^86^ ^]^ Notably, elevated levels of histamine have been detected in the blood, cerebrospinal fluid, and multiple brain regions, including the hippocampus, frontal cortex, temporal cortex, and basal ganglia of AD patients.^[^ ^65^, ^66^, ^87^, ^88^ ^]^ These findings suggest that histamine may play a significant role in AD pathogenesis. Histamine exerts its effects through four subtypes of G‐protein‐coupled receptors, known chronologically as H_1–4_R. It is crucial in regulating inflammatory responses and neurotransmission, with roles in neuroprotective and repair following nervous system injuries.^[^ ^89^ ^]^ H_4_R is distinct from the other classical histamine receptor family members, sharing very low homology with H_1–3_R,^[^ ^90^ ^]^ and mediates peripheral inflammatory diseases such as allergies, edema, asthma, and arthritis.^[^ ^89^ ^]^ H_4_R is predominantly expressed in peripheral blood cells, bone marrow, spleen, small intestine, and lungs, where it plays a critical role in modulating immune response.^[^ ^91^ ^]^ H_4_R expression has also been identified in the human cortex, mouse thalamus, hippocampus, striatum, and cortex,^[^ ^92^, ^93^ ^]^ as well as in endothelial cells,^[^ ^94^ ^]^ microglia,^[^ ^95^, ^96^ ^]^ neurons,^[^ ^92^ ^]^ but not in astrocyte.^[^ ^97^ ^]^ While the role of H_4_R within the CNS remains largely unclear, studies have shown that H_4_R antagonists exert neuroprotective effects in Parkinson‐like models by inhibiting microglia‐mediated neuroinflammation and preventing the degeneration of dopaminergic neurons in the striatum and basal ganglia.^[^ ^95^, ^96^ ^]^ However, the molecular mechanisms through which H_4_R modulates microglial activity are still not fully understood.

Microglia are long‐lived innate immune cells specialized in the phagocytic clearance of toxic stimuli. However, aging and AD can impair their phagocytic abilities, and the molecular mechanisms underlying this decline are not yet fully elucidated. Here, we demonstrate that the TGF‐β1/Smad3 signaling pathway plays a critical role in regulating microglial phagocytic ability in removing Aβ and p‐tau aggregates, and more importantly, in reversing cognitive deficits during the advanced stage of AD. It is well established that activation of the TGF‐β1/Smad3 signaling pathway mitigates proteotoxicity by enhancing microglial clearance of Aβ aggregates in AD.^[^ ^98^, ^99^, ^100^ ^]^ Conversely, Smad3 deficiency promotes α‐synuclein aggregation, which may contribute to early onset PD.^[^ ^101^ ^]^ It has been reported that activation of both H_2_R^[^ ^79^ ^]^ and H_4_R^[^ ^102^ ^]^ significantly affected the TGF‐β1 signaling pathway, specifically that H_4_R activation downregulated intracellular cAMP levels and inhibited epithelial‐to‐mesenchymal transition by suppressing TGF‐β1 signaling in lung cancer.^[^ ^102^ ^]^ Moreover, activation of TGF‐β1/Smad3 signaling in resident microglia enhanced Aβ phagocytosis.^[^ ^98^, ^100^, ^103^, ^104^ ^]^ A recent study demonstrated that Smad3 activation stimulated macrophages to phagocytose aged red blood cells and dead cells, thereby protecting the infarcted heart.^[^ ^105^ ^]^ A subset of microglia observed in the late stages of AD patients and aged AD mice, characterized by impaired Aβ phagocytic capacity, has been identified as terminally inflammatory microglia (TIM). Importantly, transcriptomic analysis predicts that TGF‐β1 signaling is severely depleted in TIM compared to DAM and homeostatic microglia.^[^ ^106^ ^]^ Consistent with these findings, we demonstrate that microglial deletion of *Hrh4* elevated cAMP levels, thereby enhancing microglial phagocytosis through activation of the TGF‐β1/Smad3 signaling pathway.

Our findings also reveal that deletion of H_4_R significantly increased intracellular cAMP levels in FACS‐sorted microglia from the cortex and hippocampus of AD mice, consistent with prior studies. H_4_R is known to decrease cAMP levels via G_αi/o_‐mediated inhibition of adenylyl cyclase.^[^ ^107^ ^]^ Most of the earlier studies on H_4_R signaling pathways have primarily focused on changes in cAMP and intracellular calcium levels. In transfected cell lines, H_4_R activation inhibited adenylyl cyclase activity, reduced cAMP production, and suppressed cAMP responsive element‐binding protein‐dependent gene transcription, while activating downstream mitogen‐activated protein kinase pathways.^[^ ^108^, ^109^ ^]^ However, in mouse bone‐marrow‐derived mast cells, H_4_R activation has been shown to elevate intracellular calcium without affecting cAMP levels.^[^ ^110^ ^]^ Additionally, H_4_R activation promoted calcium release in eosinophils and monocytes.^[^ ^111^, ^112^ ^]^ These findings suggest that the effects of H_4_R activation may vary depending on the cell type.

cAMP is known to support the integrity of the vascular endothelium in the BBB, providing protection against the progression of neurodegenerative diseases such as AD (vascular theory).^[^ ^113^ ^]^ Despite its critical role in regulating endothelial permeability, the signaling pathways involving cAMP in AD remain poorly understood. Moreover, studies investigating the impact of aging and AD on cAMP production have yielded mixed results in both animal models and clinical studies. In human and rodent, basal cAMP levels in white blood cells tend to decline with age compared to young adults.^[^ ^114^, ^115^, ^116^ ^]^ Similarly, serum cAMP levels are decreased in aged rodents relative to young adults; however, no significant differences are observed in cAMP levels within aged human cerebral microvessels.^[^ ^117^, ^118^ ^]^ In the rodent hippocampus^[^ ^119^, ^120^ ^]^ and cerebellum,^[^ ^119^, ^120^ ^]^ basal cAMP levels generally remain stable across age groups, though some exceptions have been reported.^[^ ^121^ ^]^ Notably, aged rodents show a more pronounced decline in hippocampal cAMP levels compared to young adults.^[^ ^122^ ^]^ Conversely, in the aged rodent cortex, reductions in basal cAMP levels are often observed,^[^ ^119^, ^120^ ^]^ despite some studies report no change.^[^ ^122^, ^123^ ^]^ In AD patients, decreased adenylate cyclase activity has been documented in the hippocampus,^[^ ^124^, ^125^, ^126^ ^]^ cortex,^[^ ^127^ ^]^ and cerebellum^[^ ^125^, ^127^ ^]^ when compared to healthy controls. Nonetheless, some studies have reported conflicting findings.^[^ ^128^ ^]^ One study observed elevated cAMP levels in the cerebrospinal fluid (CSF) of AD patients,^[^ ^129^ ^]^ while two others found no significant differences in CSF cAMP levels between AD patients and age‐matched controls.^[^ ^130^, ^131^ ^]^ These discrepancies suggest that changes in cyclic nucleotide levels may be localized to specific cell types, such as microglia, rather than uniformly across the entire brain.

Increasing evidence indicates that cAMP plays a critical role in microglial phagocytosis and phenotypic transformation,^[^ ^132^, ^133^ ^]^ possibly through the modulation of H_4_R and TGF‐β1 signaling pathways,^[^ ^80^, ^134^, ^135^ ^]^ as TGF‐β1 signaling is essential for microglial phagocytosis^[^ ^98^ ^]^ and the progression of AD.^[^ ^98^, ^136^, ^137^ ^]^ In non‐small‐cell lung cancer (the most common form of lung cancer), activation of H_4_R resulted in decreased cAMP levels, which suppressed TGF‐β1/Smad3 signaling. This inhibition hampered epithelial‐to‐mesenchymal transition and increased survival rates in mouse models.^[^ ^134^ ^]^ Conversely, in breast cancer cells, cAMP promoted TGFβ/Smad3‐mediated gene expression by upregulating TGF‐βR1.^[^ ^135^ ^]^ Although the correlation between cAMP and TGF‐β1/Smad3 signaling pathway is well established, we are unaware of any studies exploring the potential modulatory effects of H_4_R on the cAMP/TGF‐β1/Smad3 signaling pathway. Our findings align with recent research emphasizing the importance of microglia‐produced TGF‐β1 in maintaining microglial homeostasis and cognitive function; silencing TGF‐β1 signaling—either through ligand or receptor knockout specifically in microglia—has been shown to cause significant cognitive deficits.^[^ ^138^ ^]^ Additionally, our analysis of AD patient databases revealed a marked transcriptional down‐regulation of TGF‐βR1 in both the cortex and MTG, the latter of which has been shown to play a crucial role in episodic memory and is affected early in AD.^[^ ^83^, ^84^ ^]^ In summary, our study uncovers a previously unexplored H4R/cAMP/TGF‐β1/Smad3 signaling pathway, highlighting its potential importance in AD. This discovery warrants further research to fully understand its mechanisms and explore its therapeutic implications in AD.

## 4 Experimental Section

### Neuro‐2a Cell Culture, Aβ, and X‐Ray Irradiation Treatment

One milligram (mg) of synthetic Aβ_1–42_ peptide was dissolved in 221.7 µL of 1,1,1,3,3,3‐hexafluoro‐2‐propanol (HFIP, Sigma‐Aldrich) to prepare a 1 mm Aβ_1–42_ solution. The mixture was incubated at room temperature for 1 h to ensure complete monomerization. An aliquot containing 0.45 mg of Aβ_1–42_ was air‐dried in a fume hood for 4 h to evaporate the HFIP and then stored at −20 °C until use. The Aβ_1–42_ peptide was reconstituted in 0.1% dimethyl sulfoxide (DMSO) (Sigma‐Aldrich) and diluted with phosphate‐buffered saline (PBS) (Invitrogen) to achieve a final concentration of 1 mm Aβ_1–42_ solution, which was incubated at 4 °C for 24 h to allow Aβ oligomer aggregation.^[^ ^139^ ^]^

Neuro‐2a cells (ATCC, CCL‐131) were cultured in Dulbecco's modified Eagle medium (DMEM) (Invitrogen) supplemented with 10% fetal bovine serum (FBS, Invitrogen) and 1% penicillin/streptomycin (Invitrogen) at 37 °C in a 5% CO_2_ incubator. Cells were seeded in 96‐well plates at 3000 cells per well and allowed to adhere for 24 h. The medium was then replaced with retinoic acid medium (DMEM with 2% FBS, 1% penicillin/streptomycin, and 20 µm retinoic acid) and incubated for another 24 h to induce neuronal differentiation. The differentiated cells were positioned in the center of an X‐RAD 320 Biological Irradiator (Precision X‐ray) at a 70 cm focus‐to‐surface distance and exposed to X‐ray irradiation doses of 100, 200, 300, and 400 mGy at a dose rate of 0.08 Gy min^−1^ (320 kV, 2 mA). For control group (0 mGy), it was performed by placing the cells in the irradiator chamber without X‐ray exposure for 5 min, matching the duration of the 400 mGy dose. After irradiation, both irradiated and nonirradiated cells were incubated with Aβ (20 µm) in serum‐free medium. After 48 h, cell viability was assessed using the Cell Counting Kit‐8 (Dojindo Molecular Technologies).^[^ ^38^ ^]^

### Animals

The double‐transgenic APP/PSI mice (Stock No. 004462),^[^ ^140^ ^]^ triple‐transgenic APPswe, tauP301L, PSEN1M146V mice (3xTg‐AD, Stock No. 004807),^[^ ^42^ ^]^ and adult C57BL/6J mice (Stock No. 000664) were obtained from the Jackson Laboratory. The APP/PS1 colony was maintained by crossing WT mice with heterozygous APP/PS1 mice, producing heterozygous male offspring and gender‐matched WT littermate controls. Experiments used homozygous female 3xTg‐AD mice and age‐matched WT female mice. Genotypes were confirmed through PCR of tail biopsy genomic DNA following the primer sequences and cycling conditions recommended by Jackson Laboratory. Mice of the same gender and genotype were housed 4–5 per cage under standard conditions (23 ± 2 °C, 60–70% humidity, 12 h light/dark cycle), with free access to food and water. All procedures and euthanasia were conducted in accordance with Institutional Animal Care and Use Committee guidelines and were approved by the Animal Research Ethics Sub‐Committee at City University of Hong Kong and the Department of Health, HKSAR.

### Intracranial Injection of STZ

To induce cognitive deficits, STZ was administered via intracerebroventricular injection in adult male C57BL/6 mice as described.^[^ ^39^, ^40^ ^]^ Briefly, 12 weeks old male C57BL/6 mice were anesthetized and positioned in a stereotactic apparatus (RWD Life Science). A midline scalp incision was made, and the skin was retracted to expose the skull surface. A total of 1 µL of freshly prepared STZ (60 mg mL^−1^ in 0.05 m citrate buffer; pH 4.5) was bilaterally injected into the lateral ventricles at the following stereotactic coordinates relative to bregma: anteroposterior (AP) = ±0.3 mm, mediolateral (ML) = ±1.1 mm, and dorsoventral (DV) = +3.0 mm, using a 32G Hamilton syringe. Sham mice received an intracerebroventricular injection of an equivalent volume of citrate buffer as a vehicle control. Injections (STZ or Vehicle) were administrated on days 0 and 2, with cognitive deficits typically observed by day 21 postinjection.

### Whole‐Body X‐Ray Irradiation

Before X‐ray irradiation, adult male C57BL/6 mice (≥8 weeks old), 5.5 months old and 11.5 months old 3xTg‐AD mice, along with age‐matched WT controls, were anesthetized and secured in a ventilated mouse pie‐cage (MPC‐2; Braintree Scientific). The pie cage was positioned at the center of the X‐ray irradiator platform (X‐Rad 320, Precision X‐Ray) at a focus‐to‐surface distance of 70 cm from the irradiation source. The irradiator was calibrated with a dosimeter (UNIDOS E Dosemeter, Precision X‐Ray) prior to each session to ensure uniform X‐ray exposure. During irradiation, the machine operated at 320 kV and 2 mA, delivering a dose rate of 0.08 Gy min^−1^.^[^ ^24^, ^141^ ^]^ For the sham‐irradiated groups, mice were placed in the irradiator chamber for 37.5 and 75 s without exposure to X‐rays, matching the duration of the 50 and 100 mGy doses, respectively.

STZ mice received whole‐body X‐ray irradiation (100 mGy per session) on days 0, 7, 14, and 21 post‐STZ injections, with a total cumulative dose of 400 mGy. Additionally, both 5.5 months old and 11.5 months old 3xTg‐AD mice, as well as their age‐matched WT controls, were exposed to 50 mGy X‐ray irradiation biweekly for eight sessions, resulting in an overall dose of 400 mGy. All mice subsequently underwent neurobehavioral assessments to evaluate the therapeutic potential of low‐dose X‐ray irradiation in alleviating AD‐related cognitive deficits.

### Whole Transcriptomic Analysis Using RNA‐Sequencing (RNA‐seq)

For RNA‐seq, the hippocampus was dissected from 9 months old 3xTg‐AD mice subjected to low‐dose X‐ray irradiation or sham treatment, along with age‐matched WT controls. Total RNA was extracted using TRIzol Reagent (Takara), quantified with a NanoDrop ND‐1000 spectrophotometer, and assessed for integrity (RIN > 8.0) with an Agilent 2100 Bioanalyzer. One hundred nanograms of total RNA were depleted of rRNA using the NEBNext rRNA Depletion Kit (Human/Mouse/Rat), purified with Agencourt RNAClean XP beads, and reverse transcribed into cDNA for library preparation. Sequencing was performed on the Illumina NovaSeq 6000 platform, producing ≈75 million paired‐end reads per sample. Raw reads were aligned to the mouse reference genome GRCm38 using STAR (v2.7.8a) with default settings, and transcript abundance was quantified as transcripts per million (TPM) with RSEM (v1.3.3). Differential gene expression analysis was performed with DESeq2 (v1.30.1), using a Benjamini–Hochberg‐adjusted *p*‐value < 0.05. Variance‐stabilized counts from DESeq2 were further analyzed by principal component analysis (PCA) and hierarchical clustering.^[^ ^24^, ^48^, ^142^, ^143^ ^]^ Raw reads were aligned to the mouse reference genome GRCm38 using STAR (v2.7.8a) with default settings, and transcript abundance was quantified as TPM with RSEM (v1.3.3). Differential gene expression analysis was conducted with DESeq2 (v1.30.1), using a Benjamini–Hochberg‐adjusted *p*‐value < 0.05. Variance‐stabilized counts from DESeq2 were further analyzed via PCA and hierarchical clustering.^[^ ^24^, ^48^, ^142^, ^143^ ^]^

### Functional Enrichment Analysis

To explore the biological pathways involved in LDIR, functional enrichment analysis was performed using gene set enrichment analysis with HTSanalyzeR2 (v0.99.19). Signaling pathways and biological processes with a Benjamini–Hochberg‐adjusted *p*‐value < 0.05 were considered significant. Enrichment analyses were limited to gene sets between 5 and 500 genes to minimize bias from overly broad or narrow defined terms. The enriched GO terms and KEGG pathways were further clustered semantically using the Markov Cluster algorithm in AutoAnnotate (v1.3.2) and visualized as modular networks in Cytoscape (v3.7.2).^[^ ^24^, ^48^, ^142^, ^143^ ^]^

### In Silico Small Molecule Screening

The Library of Integrated Network‐based Cellular Signatures (LINCS) consolidated multiomics data—such as transcriptomics, proteomics, and epigenomics—across various cell types, generating over 1 million gene expression profiles in response to 44 328 bioactive small molecules. This public resource enabled in silico identification of small molecules that reproduced the transcriptional signature induced by low‐dose X‐ray irradiation. 44 DEGs from low‐dose X‐ray‐treated 3xTg‐AD mice were compared against the LINCS database. Connectivity scores, calculated using a reverse gene expression score with a range from +100 (full positive correlation) to −100 (full negative correlation), assessed similarity. Eight top candidates with scores above 90—indicating strong alignment—were prioritized.^[^ ^24^, ^48^ ^]^ Subsequently, small molecules with known efficacy in AD, reported adverse effects from chronic use, or nonavailability were excluded based on literature review and commercial availability, leaving a shortlist of candidates for functional validation by in vivo neurobehavioral testing in AD mice.

### Top‐Ranked Small Molecule Treatments

To assess the therapeutic potential of the top‐ranked small molecules, prothionamide (10 mg kg^−1^, daily oral feeding)^[^ ^144^ ^]^ and VUF6002 (10 mg kg^−1^, daily i.p. injection)^[^ ^86^ ^]^ were given to STZ‐induced sporadic AD mice for 21 days. Neurobehavioral assessments were performed to evaluate their neuroprotective effects. Additionally, to evaluate the efficacy of VUF6002 at early stages of AD, 7 months old APP/PS1 mice received either VUF6002 or a vehicle (1% carboxymethyl cellulose) daily for one month before neurobehavioral testing. For late‐stage efficacy, 12 months old APP/PS1 mice (with significant amyloid deposits)^[^ ^57^ ^]^ and 13 months old 3xTg‐AD mice (with combined amyloid and Tau pathologies)^[^ ^42^ ^]^ were treated with VUF6002 or vehicle for two months, followed by behavioral tests and histological analysis.

### Intracranial Injection of AAV and Lentivirus

To silence neuronal Hrh4 expression in vivo, a customized AAV carrying a validated shRNA targeting mouse *Hrh4* (CGAGUGCCAACUUUAGUUU, AAV2/9–*Hrh4*–shRNA)^[^ ^145^ ^]^ or a scrambled control (CCTAAGGTTAAGTCGCCCTCG, AAV2/9–scr‐shRNA) under the U6 promoter was obtained from OBiO Technology. These AAVs had titers exceeding 10^12 TU mL^−1^ to ensure efficient neuronal transduction in vivo.^[^ ^24^, ^48^, ^68^, ^70^ ^]^ Lentiviral vectors designed for microglial gene silencing under the CX3CR1 promoter were acquired from OBiO Technology.^[^ ^73^, ^74^, ^75^ ^]^ The vectors expressing shRNA targeting *Hrh4* (Lenti–*Hrh4*–shRNA), *Tgf‐βr1* (GCTGACAGCTTTGCGAATTAA, Lenti–*Tgf‐βr1*–shRNA, validated by Sigma Mission), and a scrambled sequence (Lenti–scr‐shRNA) were used for in vivo studies with titers over 10^8 TU mL^−1^ to ensure reliable microglial transduction.^[^ ^73^ ^]^ For intracranial injection, 0.5 µL of AAV or lentivirus was bilaterally injected into the cortex of mice: (AP: −0.3 mm, ML: ±2 mm, DV: −1.5 mm and AP: −2 mm, ML: ±1.2 mm, DV: −1.2 mm), the hippocampus CA1 (AP: −2 mm, ML: ±1.4 mm, DV: −2 mm), and CA3 (AP: −2.9 mm, ML: ±3 mm, DV: −3.8 mm).

### Animal Behavioral Assessment of Memory and Learning

Animals were recorded using an overhead camera (Stoelting) and analyzed with the ANY‐maze video tracking system, Version 6.05 (Stoelting Co).^[^ ^68^, ^146^ ^]^ The testing apparatuses were thoroughly cleaned with 75% ethanol between animals to remove excrement and scent residues. The researchers who performed the behavioral tests were blinded to the mouse genotypes and treatments.

### Morris Water Maze

The long‐term spatial learning and memory functions of the mice were evaluated using the MWM as described.^[^ ^68^ ^]^ The MWM was performed in a round stainless‐steel water tank measuring 150 cm in diameter and 60 cm in height, with a nonreflective interior surface to minimize light reflections. During 5 days of training, mice were placed in a circular pool filled with opacified water (nontoxic titanium dioxide, depth: 30 cm, temperature: 21 ± 1 °C) to locate a submerged hidden platform. Four distal visual cues were positioned at fixed points around the pool to aid spatial navigation. Each day, mice performed 4 trials from randomly assigned quadrants (excluding the target quadrant with the hidden platform). If a mouse failed to locate the platform within 90 s, it was gently guided to the platform and allowed to stay for 15 s. The time taken to reach the platform (latency) was recorded using ANY‐maze software (Stoelting). Twenty four hours after training, a 90 s probe trial was conducted with the platform removed, during which swimming speed, the time spent in the target quadrant, and the number of crossings over the original platform location were measured using ANY‐maze software.^[^ ^68^ ^]^

### Barnes Maze Test

The Barnes maze (diameter: 91 cm, height: 90 cm, with 20 evenly spaced escape holes around the periphery) was used to assess hippocampal‐dependent spatial memory in mice. The maze was enclosed by four black PVC boards (120 cm high, positioned 10 cm from the maze platform), with a centrally mounted 500 W floodlight providing bright illumination to motivate escape behavior. A fixed escape box was placed beneath a designated escape hole across all trials. During training, four distinct visual cues were attached to the PVC boards to assist the mice in locating the escape box. Each mouse completed three training trials per day over four consecutive days. Each trial started with the mouse placed under an opaque chamber in the maze center for 10 s, followed by 180 s of free exploration. Trials ended when the mouse entered the escape box or after the 180 s limit. Following the 4 days training, a single probe trial was performed with the escape box removed, and performance was recorded on video during this 90 s probe test. Mice that made fewer primary errors (visiting nontarget holes) and spent more time in the target quadrant were considered to have better long‐term spatial memory.^[^ ^147^ ^]^

### Y‐Maze

A Y‐shaped maze with three equal opaque arms (35 × 5 cm) was used to evaluate short‐term spatial memory in mice. The arms were labeled as zones A, B, and C, with a central hesitation zone. Each mouse was placed at the center of the Y‐maze and allowed to explore freely for 8 min before being returned to its home cages. An entry was considered valid when all four limbs were within an arm. The percentage of spontaneous alternation was calculated as the number of triads where the mouse entered all different arms (e.g., ABC, ACB, CAB, etc.) divided by the maximum possible alternations (total entries minus 2), multiplied by × 100%.^[^ ^68^ ^]^

### Novel Object Recognition

The novel object recognition test was used to evaluate the recognition memory of mice. The procedure consisted of a training phase and a test trial. During training, two identical opaque cylinders (5 cm in diameter and 10 cm in height) were placed diagonally within a 50 × 50 cm open‐field arena, equidistant from the corners. Mice were allowed to explore freely for up to 10 min, with exploration defined as nose being within 2 cm of an object, recorded using the Any‐maze system (Stoelting). The training ended when cumulative exploration reached 20 s or after the 10 min period. After a 1 h retention interval, one cylinder was replaced with a novel opaque cube (5 × 5 × 5 cm), and mice underwent a 5 min test trial. The arena and objects were cleaned thoroughly with 70% ethanol and dried between sessions to eliminate olfactory cues. The recognition index was calculated as: (exploration time of the novel object/(exploration time of the novel + familiar objects)) × 100%.^[^ ^148^ ^]^

### Western Blot Analysis

Western blot analysis of total protein extracts from the hippocampus and cortex of AD mice was performed as described.^[^ ^24^, ^70^, ^149^, ^150^, ^151^ ^]^ Briefly, hippocampal and cortical tissues were lysed, and protein concentrations were measured using a BCA assay (Pierce). For each sample, 20 µg of protein lysate was separated on a 14% Tris‐Glycine Mini gel (Invitrogen). The PVDF membrane was blocked with 5% bovine serum albumin for 1 h and then incubated overnight at 4 °C with primary antibodies: anti‐6E10 (1:1000; Biolegend), anti‐p‐tau Ser202 + Thr205 (1:1000; Thermo Fisher Scientific), anti‐Total Tau (1:1,000; Santa Cruz), anti‐ TGF‐βR1 (1:200, Santa Cruz), anti‐CD68 (1:1000, Abcam), anti‐BACE1 (1:1000, Abcam), anti‐ADAM10 (1:1000, Cell Signaling Technology), and anti‐H_4_R (1:1000, Biorbyt). The bands were visualized using the Immobilon Western Chemiluminescent HRP Substrate Kit (Millipore). The membranes were then stripped and reprobed with anti‐β‐actin (1:20 000; Cell Signaling Technology) as loading controls. Band quantification was performed with ImageLab software (Bio‐Rad).

### Immunohistochemistry

Adult mice were transcardially perfused with PBS followed by 4% paraformaldehyde. The entire brain was extracted, postfixed, and cryopreserved in OCT compound (Tissue‐Tek).^[^ ^24^, ^48^, ^68^, ^70^, ^141^ ^]^ Coronal brain sections (40 µm thick) were blocked with 0.5% bovine serum albumin and 0.1% Triton X‐100 (Sigma‐Aldrich) in PBS, then incubated overnight at 4 °C with primary antibodies: anti‐6E10 (1:200; Biolegend), anti‐p‐tau Ser202 + Thr205 (1:50; Thermo Fisher), anti‐Iba1 (1:500; Wako), anti‐CD68 (1:200; Abcam), anti‐CD11b (1:200; Abcam), and anti‐p‐Smad3 (1:200; Abcam). After three washes with PBS, cryosections were incubated with appropriate secondary antibodies. Images were captured at 20× magnification using a Nikon A1HD25 confocal microscope, then stitched and maximally projected with NIS‐Element's software (Nikon). Four coronal brain sections per mouse and 3–5 mice per treatment group were analyzed.

### 3D Reconstruction of Microglia, Phagosomes, and Aβ Plaques

The immunoreactivity of microglia (Iba1, 1:500, Wako), lysosomes (CD68, 1:200, Abcam), Aβ plaques (6E10, 1:500, Biolegend), and p‐tau (1:50, Thermo Fisher) were labeled with their respective primary antibodies. A series of z‐stack images were acquired using a Nikon A1HD25 confocal microscope. To quantify Aβ internalization ratio, the colocalization volume of 6E10‐positive Aβ plaques within microglial lysosomes (Iba1^+^CD68^+^) was normalized to total Aβ plaque volume.^[^ ^57^ ^]^ Similarly, for p‐tau internalization, the colocalization volume of p‐tau (Ser202 Thr205^+^) within microglial phagosomes (Iba1^+^CD68^+^) was normalized to total p‐tau volume.^[^ ^152^ ^]^ All imaging parameters, background subtraction, and thresholds settings were kept consistent across all samples and treatment groups.

### Isolation of Microglia and Neurons from Adult Mice by FACS Analysis

Mice were perfused with ice‐cold PBS to remove blood. The hippocampus and cortex were cut into pieces and homogenized in PBS using a Dounce homogenizer, then filtered through a 70 µm cell strainer. The resulting brain homogenate was resuspended in 70% Percoll, overlaid with 40% Percoll, and centrifuged at 2000 rpm for 30 min. The upper layer containing cell debris and myelin was discarded. The 40% Percoll layer and the interlayer between 40% and 70% Percoll, which contained microglia and neurons, were collected for FACS analysis.^[^ ^153^ ^]^

For microglia isolation, live and dead cells were stained using the Zombie Green Fixable viability kit (1:1000, BioLegend).^[^ ^154^ ^]^ The isolated mononuclear cell was pretreated with CD16/CD32 FC blocker (1:200, BD Biosciences) to prevent nonspecific antibody binding, and then incubated with CD11b‐PE (1:100, BD Biosciences) and CD45‐APC/Cyanine 7 (1:100, BioLegend). The myeloid cells were identified as resident microglia cells (CD11b^+^CD45^low^), while infiltrating monocytes were classified as (CD11b^+^CD45^high^).^[^ ^81^ ^]^ Resident microglia were sorted using a Sony SH800Z cell sorter for downstream analysis.

For neuron isolation, live and dead cells were stained using the Zombie Green Fixable viability kit (1:1000). The isolated mononuclear cells were pretreated with 50% ice‐cold ethanol on ice for 15 min to permeabilize the cells. Following permeabilization, the mononuclear cells were centrifuged at 5400 rpm for 5 min and resuspended in ice‐cold PBS. The solution was then incubated on ice for 1 h with anti‐NeuN antibody (1:100, Sigma‐Aldrich), anti‐mouse Alexa Fluor 647 secondary antibody (1:100, Thermo Fisher Scientific), and Hoechst 33258 (1:1000, Sigma‐Aldrich). NeuN and Hoechst 33258 double‐positive cells (Hoechst^+^NeuN^+^) were identified as neurons and sorted using a Sony SH800Z cell sorter for further analysis.

### Quantification of Microglial cAMP Concentration

Two weeks after lentivirus injection, microglia were isolated from the brains of 12.5 months old APP/PS1 mice using FACS. To prevent cAMP degradation, the microglia were lysed immediately postharvest in a cell lysis buffer containing 0.1 n hydrochloric acid. The homogenate was centrifuged at 10 000 × *g* for 10 min to remove insoluble debris, and the supernatant was neutralized with 1 n NaOH at 1:10 v/v ratio relative to the total sample volume. The cAMP levels were measured using the Mouse/Rat cAMP Parameter Assay Kit (R&D Systems) according to the manufacturer's instructions and normalized to total protein concentration.^[^ ^155^ ^]^

### In Vivo Microglial Phagocytosis of Aβ Assay

Aged APP/PS1 mice were intraperitoneally injected with methoxy‐X04 (Tocris) at 10 mg kg^−1^ (dissolved in 50% DMSO and 0.9% NaCl) to label insoluble Aβ fibrils in the brain. Three hours post‐injection, the mice were perfused with ice‐cold PBS, and their brains were harvested. The hippocampi and cortices from two mice within the same treatment group were pooled and homogenized. Microglia were labeled with CD11b‐PE (1:100, BD Biosciences) and CD45‐APC/Cyanine 7 (1:100, BioLegend). Flow cytometry analysis on the Beckman Coulter CytoFLEX quantified the proportion of CD11b^+^CD45^low^ microglia containing methoxy‐X04‐positive Aβ fibrils (MeX04^+^), reflecting microglial phagocytic activity of Aβ.^[^ ^81^, ^156^ ^]^

### ELISA Quantification of Human Aβ_1–42_ and Human Aβ_1–40_

The hippocampus or cortex tissues were sonicated in ice‐cold PBS containing a protease inhibitor, then centrifuged at 14 000 *g* for 20 min. The soluble Aβ fractions from the supernatant were collected for protein assay using the BCA assay. The concentrations of human Aβ_1–42_ and Aβ_1–40_ were measured with the ELISA assay kit (Cusabio) following the manufacturer's instructions. The concentrations of human Aβ_1–42_ or Aβ_1–40_ were normalized to the total protein concentrations for each sample.^[^ ^11^ ^]^

### Detection of VUF6002 in Mouse Brain by High‐Performance Liquid Chromatography (HPLC)–MS/MS Analysis

To evaluate the BBB permeability of VUF6002, mouse brains were collected at various time points (0.25, 0.5, 1, 2, 4, 8, and 12 h, *n* = 3 per time point) and analyzed via HPLC–MS/MS as described.^[^ ^70^ ^]^ Fresh brain samples were weighed equally and mixed with an equal volume of HPLC‐grade methanol, then sonicated for 30 min in an ultrasonic bath (Grant). After centrifugation at 14 000 rpm for 30 min at 20 °C, the supernatant was collected for LC–MS/MS analysis.

Qualitative and quantitative determination of VUF6002 was performed using an Agilent 1200 Series HPLC system (Agilent Technologies Inc., Santa Clara, CA, USA) coupled with an Applied Biosystems (ABI, USA) 2000 QTrap triple quadrupole linear ion trap mass spectrometer (MS/MS) equipped with a Turbo Ion source for electrospray ionization, operated with Analyst software version 1.5.2 (Applied Biosystems, Canada). One microliter of sample was injected via the autosampler. Chromatographic separation employed a Waters XBridge BEH HILIC Column (130 Å, 3.5 µm, 2.1 mm × 50 mm) with a Waters XBridge BEH HILIC 2.5 µm guard column (2.1 mm × 5 mm). The analyte was separated isocratically at 200 µL min^−1^, using a mixture of 0.1% acetic acid and 50 mm ammonium acetate in Milli‐Q water and methanol in a 3:97 v/v ratio. The retention time for VUF6002 was 0.74 min.

### qPCR

Total RNA from the hippocampus was isolated using TRIzol Reagent (Takara) supplemented with 1 mm dithiothreitol. The extracted RNA was reverse‐transcribed into cDNA buy using the Prime Script RT reagent kit (Takara). The qPCR assays were performed in duplicate using the KAPA SYBR Fast qPCR Kit (KAPA) on a QuantStudio 7 Pro real‐time PCR system (Applied Biosystems). The housekeeping gene *Gapdh* was used as a loading control. Gene expression levels were normalized to *Gapdh* and the fold change was calculated using the 2^−ΔΔCt method, relative to age‐matched vehicle‐treated control mice.^[^ ^157^ ^]^

### Gene Expression Omnibus (GEO) Database Analysis

To investigate the expression dynamics of *Tgf‐βr1* (Homo sapiens; RefSeq: NM_004612.4) in AD, RNA‐seq and microarray datasets were retrieved from the GEO https://www.ncbi.nlm.nih.gov/geo/). Preprocessed Series Matrix Files (normalized expression matrices) along with their corresponding annotation platforms (GPL files) were downloaded for integrated analysis. The expression levels of *Tgf‐βr1* in the middle temporal gyrus (GSE5281, platform: GPL570) and cortex (GSE15222, platform: GPL570) in AD patients were normalized against healthy controls within the same brain regions by calculating the relative log2 fold change.

### Statistical Analysis

Experimental data were presented as means ± standard error of the mean (SEM). Differences in the Barnes maze and water maze training tests were analyzed using two‐way ANOVA, followed by Tukey's post hoc tests for multiple pairwise comparisons. Comparisons between two treatment groups were performed using Student's *t*‐test. For other behavioral tests and assay parameters, one‐way ANOVA with Tukey's post hoc tests was used. GEO database analyses were conducted with the Mann–Whitney *U* test. All statistical analyses were conducted using GraphPad Prism software version 9.0, with a *p*‐value < 0.05 considered statistically significant.

## Conflict of Interest

The authors declare no conflict of interest.

## Author Contributions

Y.‐J.X. performed the cell culture, X‐ray irradiation, small molecule administration, AAV and lentivirus injections, animal neurobehavioral assessment, Western blot analysis, immunohistochemistry and analysis, and flow cytometry. T.W. and X.W. performed the bioinformatic analysis on RNA‐seq. L.T.‐L.L. and C.‐C.K. performed the pharmacokinetics study of VUF6002. C.H.E.M. conceived the project. Y.‐J.X. and C.H.E.M. wrote the paper.

## Supporting information



Supporting Information

## Data Availability

The data that support the findings of this study are available from the corresponding author upon reasonable request. Public RNA‐sequencing data are available in the NCBI GEO under accession no.: GSE246670.
