# Supplementary material for: Microglial Deletion of Hrh4 Alleviates Alzheimer's Disease Pathologies by Enhancing Microglial Phagocytosis of Amyloid‐β and Tau
Source: Adv Sci (Weinh). 2025 Oct 24;13(2):e05421. doi: 10.1002/advs.202505421 (PMC12786338; doi:10.1002/advs.202505421)
Supplement: Supplementary file 1 — Supporting Information [file ADVS-13-e05421-s001.pdf]

Supporting Information

Microglial deletion of *Hrh4* alleviates Alzheimer's disease pathologies by enhancing microglial phagocytosis of amyloid-β and tau

Yi-Jun Xu[1], Tan Wu[2, 3], Larry Tso-Lun Lo[4], Chi-Chiu Ko[4], Xin Wang[2, 3], Chi Him Eddie Ma[1,*]

[1] Department of Neuroscience, City University of Hong Kong, Tat Chee Avenue, Hong Kong SAR, China

[2]Department of Surgery, The Chinese University of Hong Kong, Hong Kong, China

[3]Department of Biomedical Sciences, City University of Hong Kong, Tat Chee Avenue, Hong Kong SAR, China

[4]Department of Chemistry, City University of Hong Kong, Tat Chee Avenue, Hong Kong SAR, China

* Correspondence: Prof. Chi Him Eddie Ma

Email: eddiema@cityu.edu.hk

Phone: (852)-3442-9328

Fax: (852)-3442-0549

Number of Supplemental Figures: 15

Figure S1

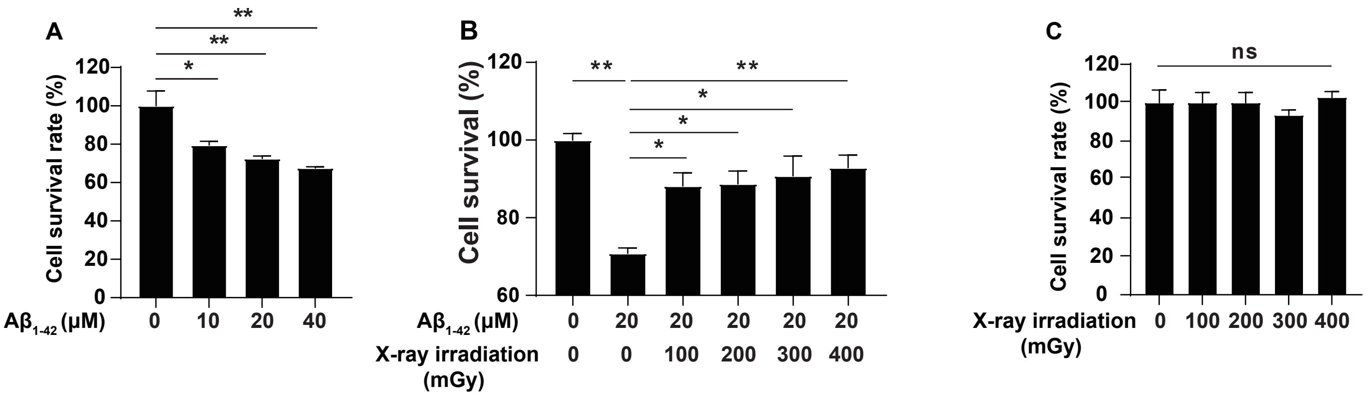

Fig. S1. LDIR attenuates Aβ$_{1\text{-}42}$-induced neurotoxicity in differentiated mouse Neuro-2a (N2a) cells. N2a cells were cultured in a DMEM for 24 h, differentiated with retinoic acid for 24 h, and then exposed to LDIR at various doses (100-400mGy) prior to Aβ$_{1\text{-}42}$ peptides (20 μM) challenge for 48 h in a serum-free culture medium. Cell viability tests were performed using CCK-8 assay. **(A)** Treatment with 0–40 μM Aβ$_{1\text{-}42}$ peptides for 48 hours caused a marked reduction in cell viability compared to untreated controls. **(B)** LDIR (100-400 mGy) markedly mitigated Aβ$_{1\text{-}42}$-induced cytotoxicity, with the maximal protective efficacy observed at 400 mGy. **(C)** LDIR (100-400 mGy) alone did not affect the cell survival of N2a cells. Mean ± SEM (n = 3 per group). * P < 0.05; ** P < 0.01; ns: not significant; one-way ANOVA followed by Tukey's post hoc test (A-C).

Figure S2

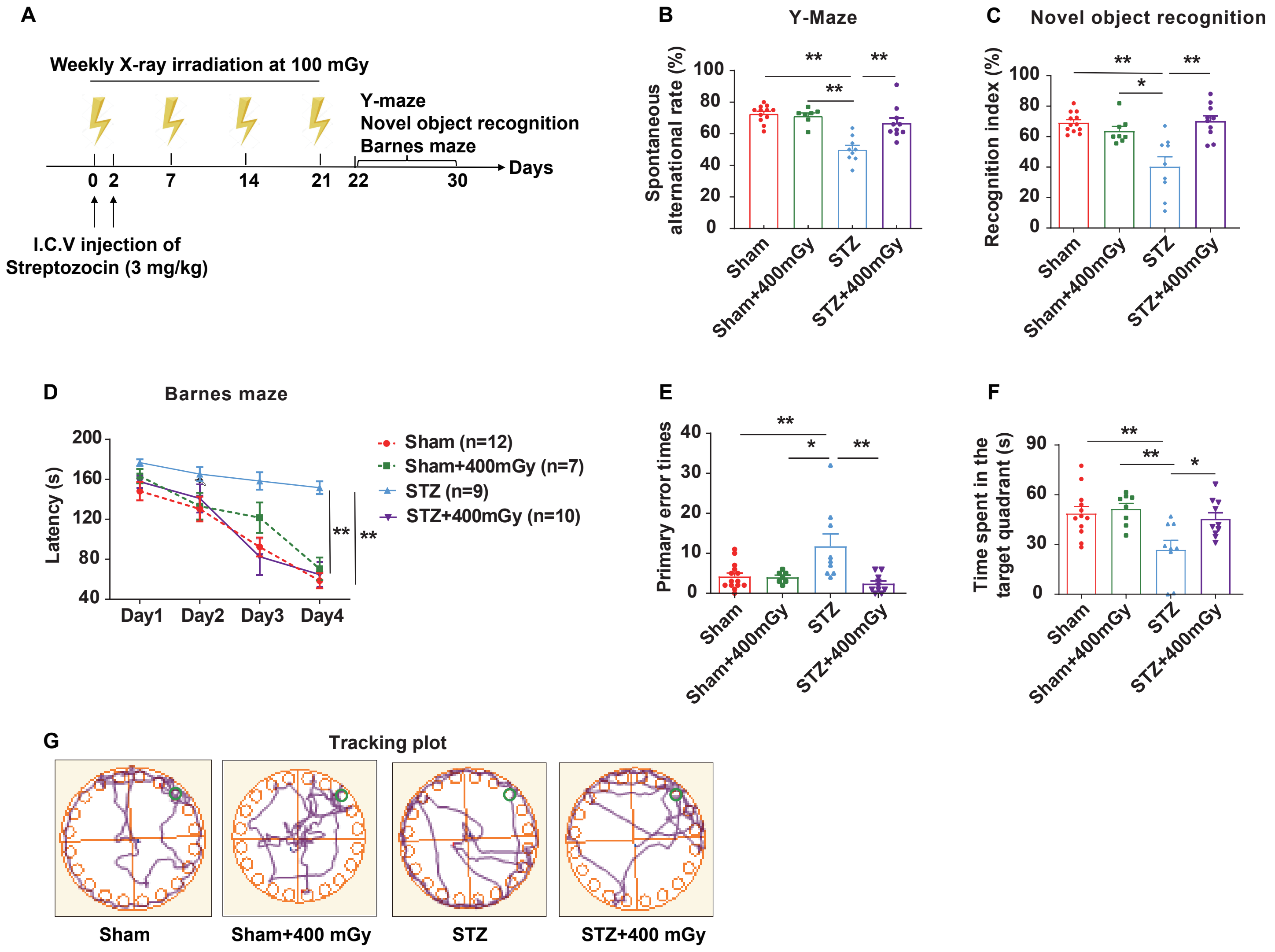

Fig. S2. LDIR reverses memory deficits of the streptozotocin (STZ)-induced sporadic AD mouse model. (A) Adult male C57BL/6 mice received intracerebroventricular (ICV) injections of STZ (3 mg/kg) on days 0 and 2. STZ-injected mice (STZ-mice) were then subjected to weekly whole-body X-ray irradiation (100 mGy) on days 0, 7, 14, and 21, for a total cumulative dose of 400 mGy, followed by neurobehavioral assessments. **(B)** LDIR at 400 mGy restored working memory performance in STZ-mice to levels comparable to sham controls in the Y-maze test. **(C)** STZ-mice failed to recognize the novel object in the novel object recognition test, but LDIR at 400 mGy recovered their recognition ability to sham-equivalent levels. **(D)** STZ-mice showed long-term spatial memory deficits by taking longer to locate the escape box in the Barnes maze; LDIR-treated mice exhibited similar latencies to sham controls. **(E–G)** Compared to sham mice, STZ-mice made more errors (E), spent significantly less time in the target quadrants (F), and displayed more disorganized search paths (G) in the probe trial when locating the escape hole (green circles). LDIR at 400 mGy completely reversed these spatial memory deficits, with performance metrics comparable to sham controls. LDIR-treated sham mice exhibited no significant behavioral differences compared to uninjured sham mice across all neurobehavioral assessments. Mean ± SEM (n = 7–12 per group). * $P < 0.05$; ** $P < 0.01$; one-way ANOVA followed by Tukey's post hoc test (B–C and E–F); two-way repeated measures ANOVA followed by Tukey's post hoc test (D).

Figure S3

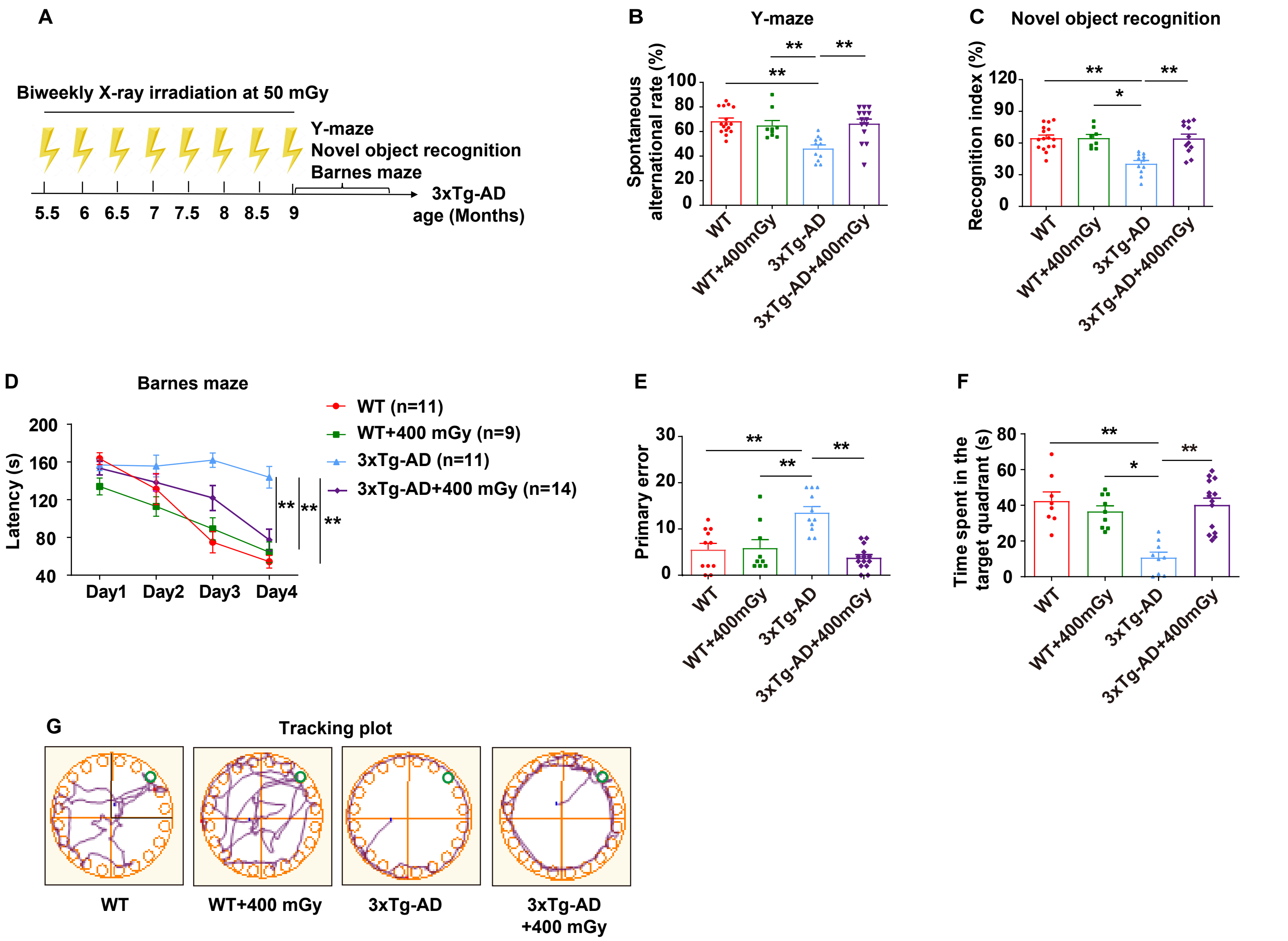

Fig. S3. LDIR alleviates cognitive deficits in 9-month-old 3xTg-AD mice. (A) Female 3xTg-AD mice aged 5.5 months and age-matched wildtype (WT) controls received biweekly LDIR treatment (50 mGy per dose) for 14 weeks, reaching a total dose of 400 mGy (8 doses) by 9 months of age. Cognitive function was assessed at the end of treatment using neurobehavioral tests. **(B)** Sham-irradiated 3xTg-AD mice exhibited working memory deficits, evidenced by reduced spontaneous alternation in the Y-maze compared to WT controls. LDIR treatment restored working memory performance in 3xTg-AD mice to levels comparable with WT. **(C)** In the novel object recognition test, 3xTg-AD mice showed impaired recognition ability, indicated by a lower percentage of time exploring the novel object. LDIR treatment rescued recognition memory, bringing it to WT levels. **(D)** LDIR improved spatial memory in 3xTg-AD mice, as reflected by shorter latency during Barnes maze training. **(E-G)** LDIR reversed spatial memory deficits by decreasing primary errors (E) and increasing exploration of the target quadrant (F–G). The green circle indicates the location of the escape box. LDIR did not affect the performance of WT mice across all neurobehavioral assessments. Mean ± SEM (n = 9 - 14 per group). * $P < 0.05$; ** $P < 0.01$; one-way ANOVA followed by Tukey's post hoc test (B–C, and E–F); two-way repeated-measures ANOVA followed by Tukey's post hoc test (D).

Figure S4

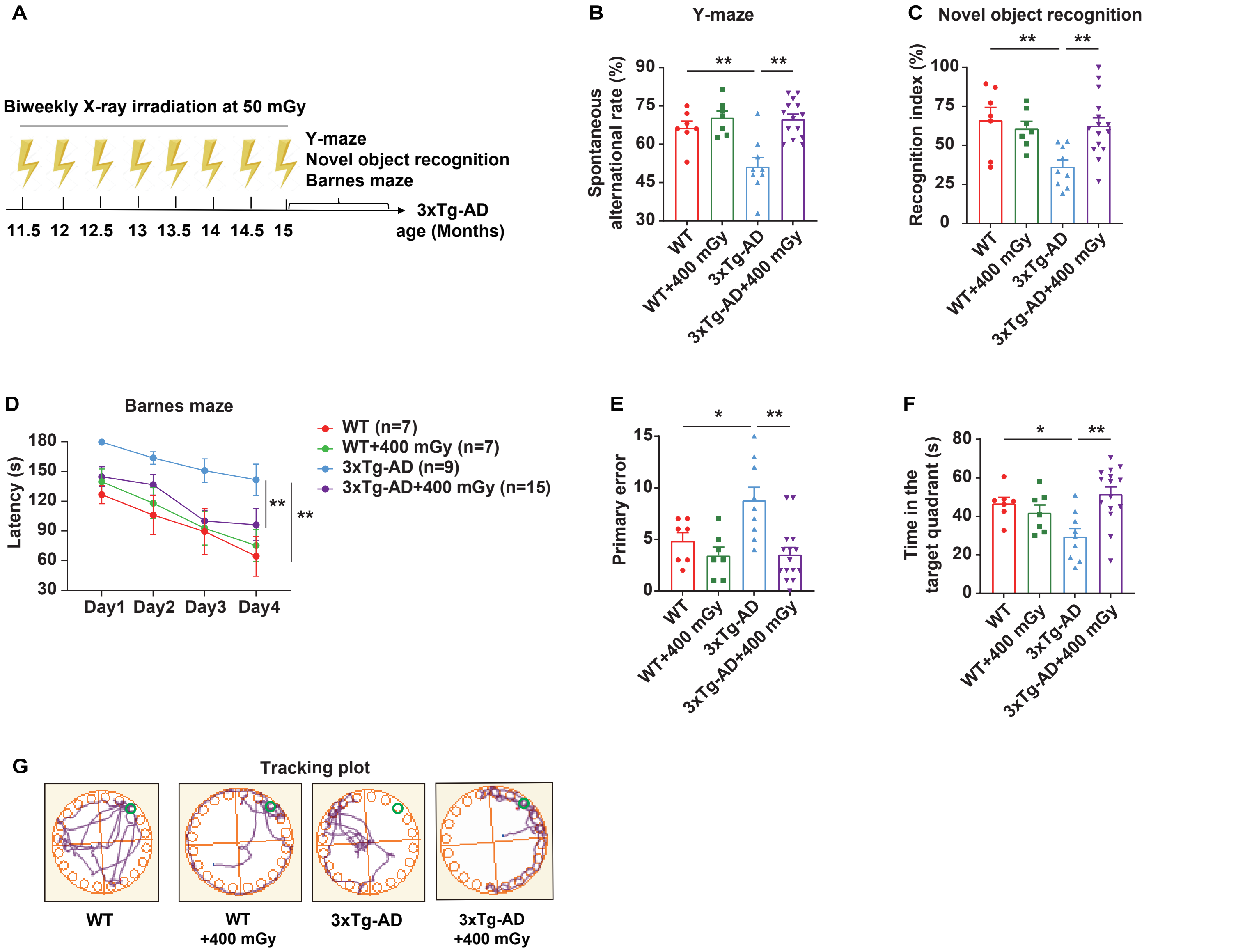

Fig. S4. LDIR alleviates cognitive deficits in 15-month-old 3xTg-AD mice. (A) The 11.5-month-old 3xTg-AD mice and their age-matched wildtype (WT) controls received whole-body X-ray irradiation at 50 mGy (or sham) every two weeks until they reached 15 months (a total of 8 doses with cumulative dose of 400 mGy), followed by neurobehavioral testing. **(B)** TThe 3xTg-AD mice exhibited a significant reduction in the spontaneous alternation rate in the Y-maze test, indicating short-term working memory deficits. LDIR treatment improved their spatial working memory, bringing performance to levels comparable with WT mice. **(C)** LDIR-treated 3xTg-AD mice showed restored recognition ability, demonstrated by a significant increase in exploration time of the novel object and discrimination index similar to WT controls. **(D)** LDIR significantly shortened the latency to find the escape box in the Barnes maze compared to sham-irradiated 3xTg-AD mice. **(E-G)** During the Barnes maze probe trial, sham-irradiated 3xTg-AD mice made more primary errors (E) and spent less time in the target quadrant (F) than age-matched WT controls. LDIR reversed these deficits, resulting in fewer errors and increased time in the target quadrant (G). The green circle indicates the location of the escape box. LDIR did not induce behavioral changes in WT mice across all neurobehavioral assessments. Mean ± SEM (n = 7–15 per group). * $P < 0.05$; ** $P < 0.01$; one-way ANOVA followed by Tukey's post hoc test (B-C and E-F); two-way repeated measures ANOVA followed by Tukey's post hoc test (D).

Figure S5

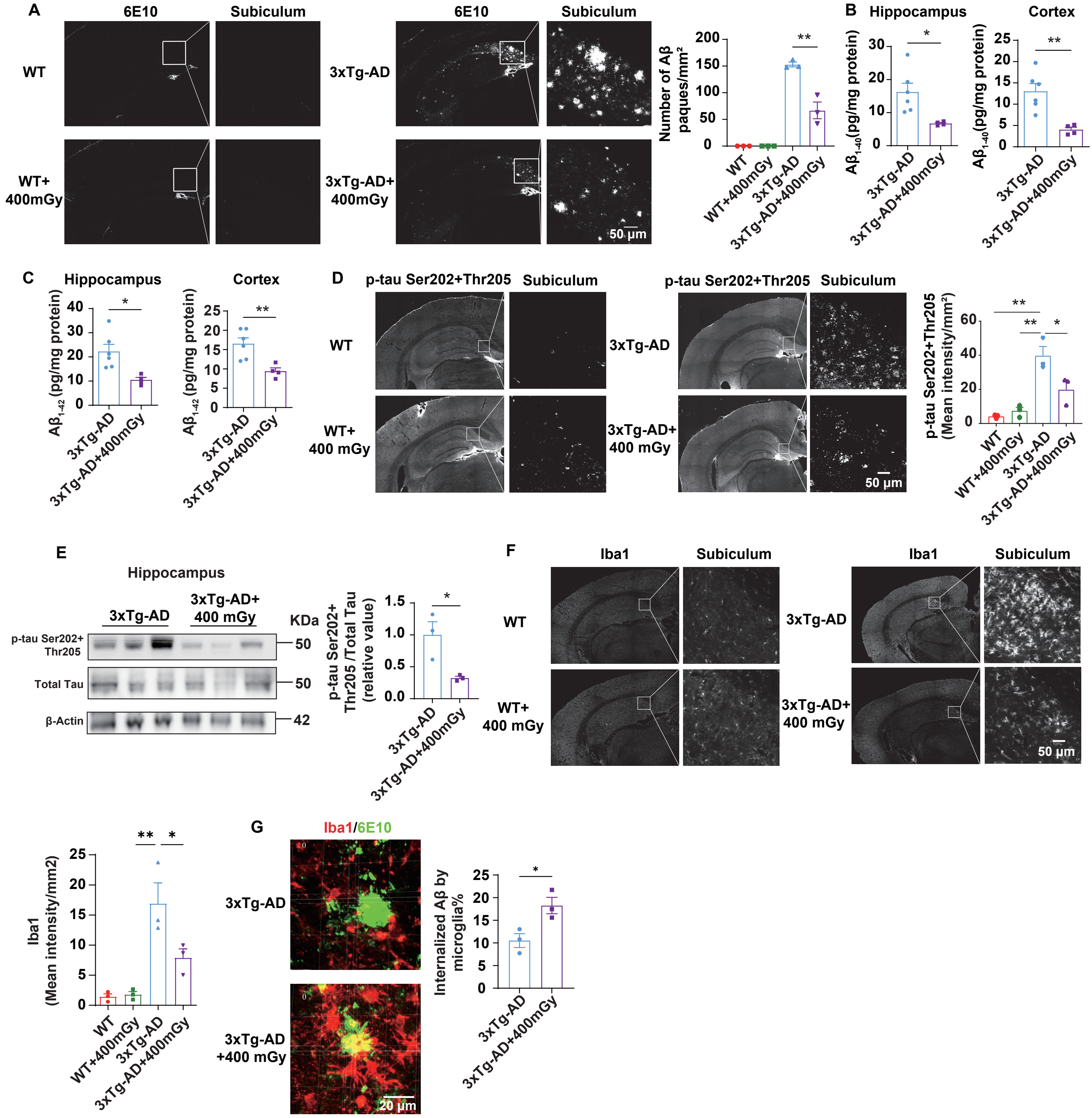

Fig. S5. LDIR reduces Aβ deposition, hyperphosphorylated tau (p-tau) and microgliosis in the subiculum in 15-month-old 3xTg-AD mice. (A) LDIR significantly decreased the immunoreactivity of 6E10-labeled Aβ plaques in the hippocampal subiculum of aged 3xTg-AD mice. Scale bar: 200 μm. (n=3 per group). **(B-C)** The concentrations of soluble human $A\beta_{1-40}$ and $A\beta_{1-42}$ in hippocampal and cortical lysates were quantified using ELISA assays following LDIR treatment. (n = 4 - 6 per group). **(D)** Representative photomicrographs showing reduced p-tau at Ser202 and Thr205 in the subiculum of LDIR-treated 3xTg-AD mice. Scale bar: 50 μm. (n=3 per group). **(E)** Western blot analysis using anti-AT8 antibodies revealed a significant reduction in p-tau in the hippocampus of LDIR-treated mice, with p-tau levels normalized to Total Tau. Each lane represents one sample from an individual mouse. (n = 3 per group). **(F)** Representative confocal photomicrographs illustrating decreased microgliosis, indicated by Iba1 immunoreactivity in the hippocampal subiculum of 15-month-old 3xTg-AD mice following LDIR. Iba1 intensity was measured with NIS-Elements software (Nikon). Scale bar: 50 μm. (n = 3 per group). **(G)** Three-dimensional surface reconstruction analysis of Iba1-positive microglia (red) colocalized with 6E10-positive Aβ plaques (green) in the subiculum. The colocalized volume was normalized to the Aβ volume in vehicle- and LDIR-treated mice (n=64 and 80 plaques, respectively). Scale bar: 20 μm. (n = 3 per group). Mean ± SEM. * $P < 0.05$; ** $P < 0.01$. one-way ANOVA followed by Tukey's post hoc test (A, D, and F); Student's t-test (B, C, E, and G).

Figure S6

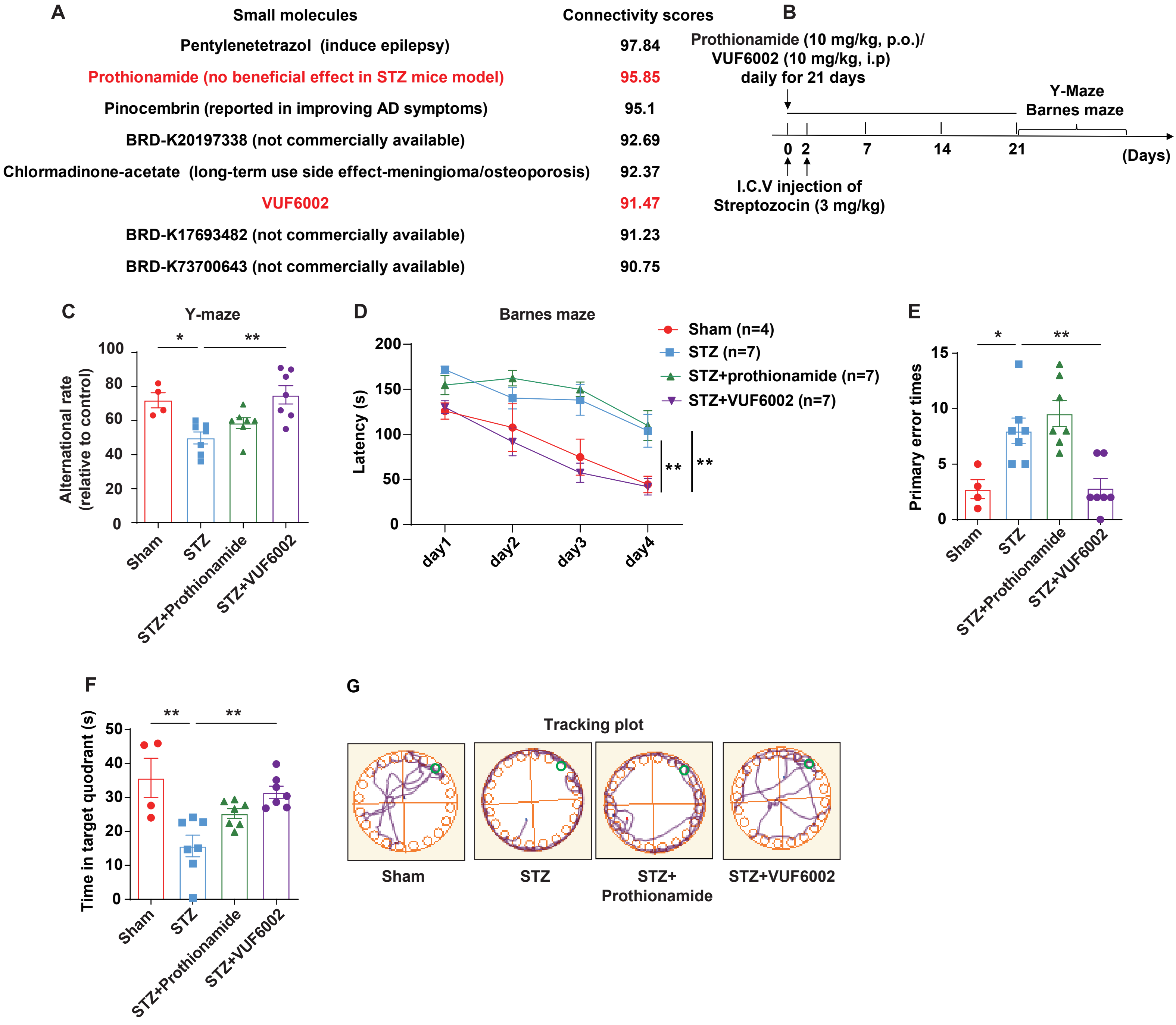

Fig. S6. In silico screening of small molecule VUF6002 mimics the beneficial effects of LDIR. (A) LDIR treatment induced 44 differentially expressed genes (DEGs; adjusted p < 0.05, Benjamini-Hochberg) in 3xTg-AD mice. These DEGs were queried against the LINCS (Library of Integrated Network-based Cellular Signatures) database. Using pattern-matching algorithms, eight small molecules with high connectivity scores (> 90) were identified as potential mimics of LDIR's beneficial effects. Excluded from the list were compounds with known adverse effects from chronic treatment (pentylenetetrazol, chlormadinone-acetate), compounds with reported therapeutic efficacy in AD (pinocembrin), and those not commercially available (BRD-K20197338, BRD-K17693482, BRD-K73700643). **(B)** TTo evaluate the protective effects, STZ mice were treated with prothionamide (10 mg/kg, p.o.) or VUF6002 (10 mg/kg, i.p.) for 21 days, followed by neurobehavioral assessments.**(C)** The Y-maze test revealed a significant improvement of working memory in VUF6002-treated STZ-mice. **(D-G)** Spatial memory assessed via the Barnes maze revealed no significant differences between sham and VUF6002-treated STZ-mice in escape latency during training (D), the number of primary errors (E), or the time spent in the target quadrant during the probe trial (F-G) (n = 4 - 7 per group). Prothionamide exhibited no therapeutic efficacy in this model. Mean ± SEM. * $P < 0.05$; ** $P < 0.01$. one-way ANOVA followed by Tukey's post hoc test (C and E–F); two-way repeated-measures ANOVA followed by Tukey's post hoc test (D).

Figure S7

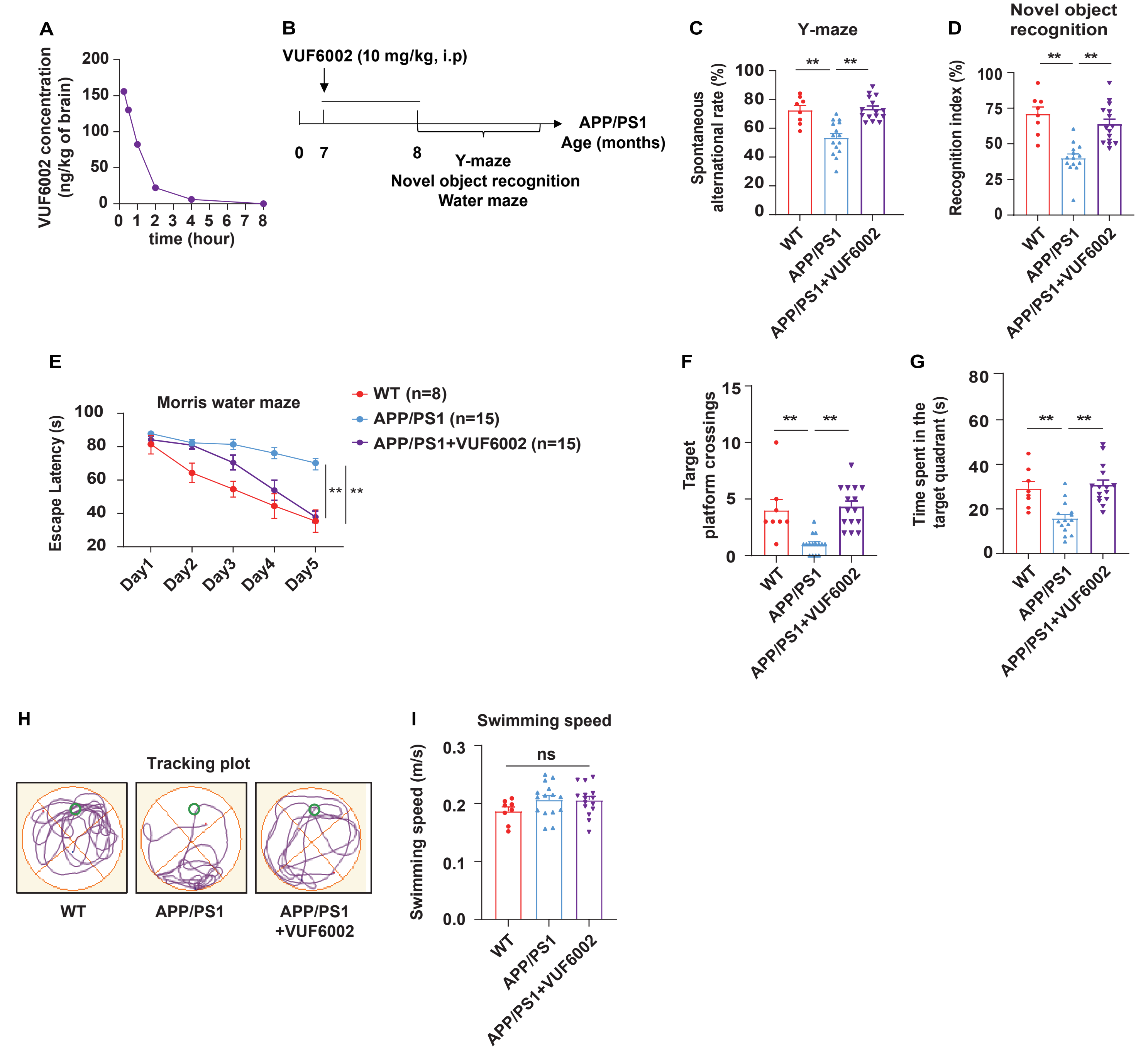

Fig. S7. VUF6002 reverses memory deficits of 8-month-old APP/PS1 mice. (A) Pharmacokinetic analysis of VUF6002 in the adult mouse brain was performed using HPLC-MS/MS, with concentrations at each time point expressed as mean (n = 3). **(B)** APP/PS1 mice were treated daily with VUF6002 at 10 mg/kg (or 1% carboxymethyl cellulose [CMC] as vehicle control) from 7 to 8 months of age. **(C)** VUF6002 treatment restored working memory in APP/PS1 mice to levels comparable to wildtype (WT) controls in the Y-maze test. **(D)** In the novel object recognition test, APP/PS1 mice displayed deficits by spending less time exploring the novel object; these deficits were reversed with VUF6002 treatment. **(E)** VUF6002 significantly reduced the latency to find the hidden platform during the 5-day training of the Morris water maze. (F–H) In the probe trial (platform removed), VUF6002-treated APP/PS1 mice showed increased platform crossings **(F)** and spent more time in the target quadrant **(G–H)** compared to vehicle-treated mice. **(I)** There was no difference in swimming speeds across groups during the probe trial. (n = 8 – 15 per group). Mean ± SEM. * $P < 0.05$; ** $P < 0.01$, ns: not significant; one-way ANOVA followed by Tukey's post hoc test (C-D, F-G, and I); two-way repeated-measures ANOVA followed by Tukey's post hoc test (E).

Figure S8

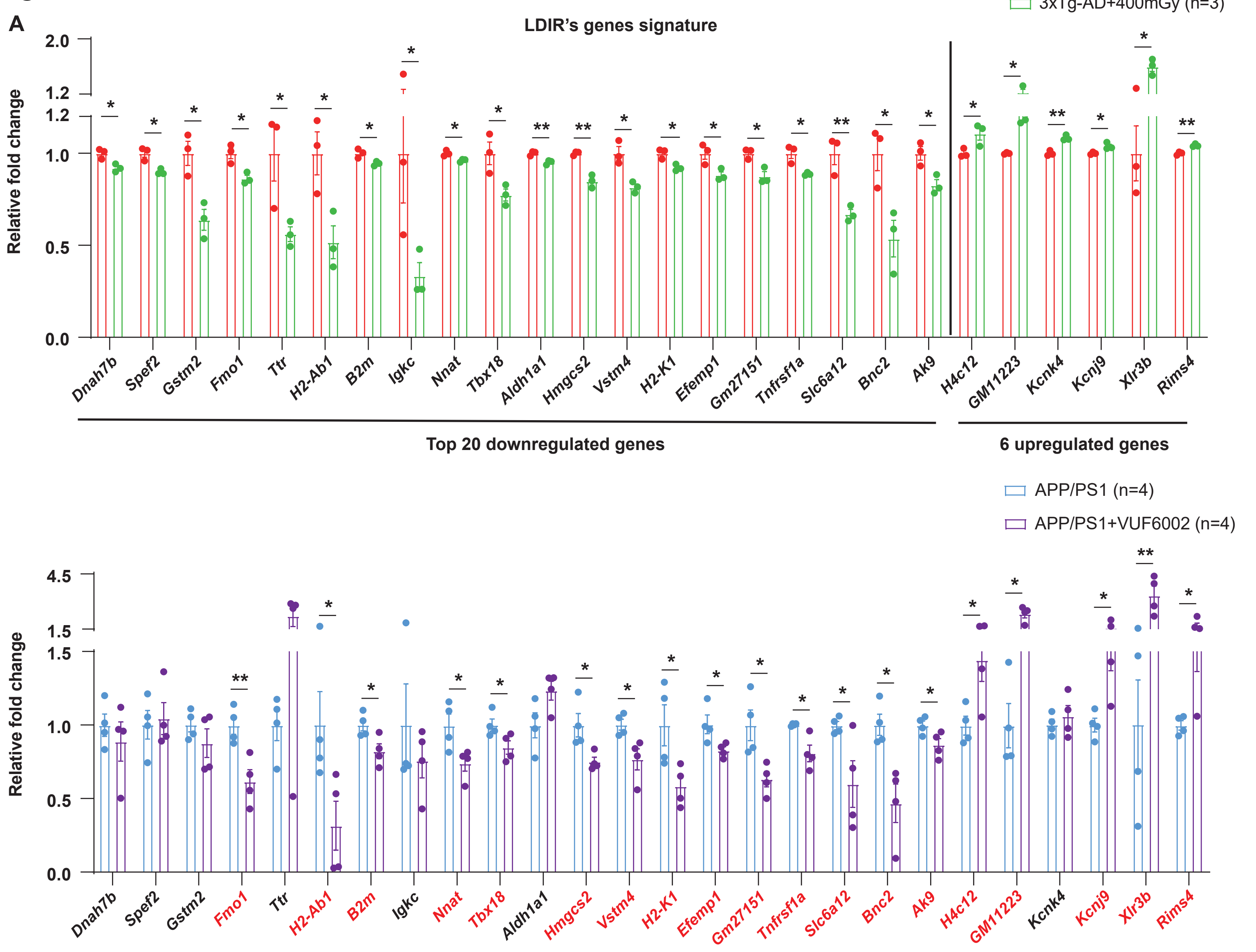

B

Upregulated genes	GO TERM_BP_DIRECT	Biological process
H4c12	319160	nucleosome assembly
Kcnj9	16524	regulation of presynaptic membrane potential
Xlr3b	574437	positive regulation of synapse assembly
Rims4	241770	regulation of synapse organization, regulation of synaptic vesicle exocytosis

Downregulated genes	GO TERM_BP_DIRECT	Biological process
Fmo1	14261	response to lipopolysaccharide
H2-ab1	14961	adaptive immune response, antigen processing and presentation of exogenous peptide antigen via MHC class II
B2m	12010	immune response, antigen processing and presentation of exogenous peptide antigen via MHC class II, amyloid fibril formation
Nnat	18111	positive regulation of insulin secretion
Tbx18	76365	cell differentiation
Ak9	633979	UDP/CDP biosynthetic process
Bnc2	242509	regulation of DNA-templated transcription
Slc6a12	14411	neurotransmitter transport
Tnfrsf1a	21937	positive regulation of neuron apoptotic process, positive regulation of inflammatory response
Efemp1	216616	positive regulation of cell population proliferation
H2-K1	14972	T cell mediated cytotoxicity, immune response
Vstm4	320736	endothelial cell proliferation
Hmgcs2	15360	cellular response to lipopolysaccharide

Fig. S8. VUF6002 mimics LDIR's transcriptomic and biological effects. (A) The gene signature of LDIR includes 6 upregulated and 38 downregulated differentially expressed genes (DEGs) in young 3xTg-AD mice, associated with its therapeutic effects against AD. Expression levels of all 6 upregulated genes and the top 20 downregulated genes (ranked by adjusted p-value) were validated by qPCR in the hippocampus of VUF6002-treated young APP/PS1 mice. VUF6002 induced similar expression changes in 19 out of 26 genes (73.5%), overlapping with LDIR's signature (highlighted in red), confirming a shared transcriptional response. Mean ± SEM. **(B)** Gene set over-representation analysis (GSOA) revealed that VUF6002 significantly enriched pathways related to nucleosome assembly and synaptic regulation while suppressing immune response pathways, demonstrating substantial functional overlap with LDIR's biological effects. Mean ± SEM. * $P < 0.05$; ** $P < 0.01$, Student's t-test (A).

Figure S9

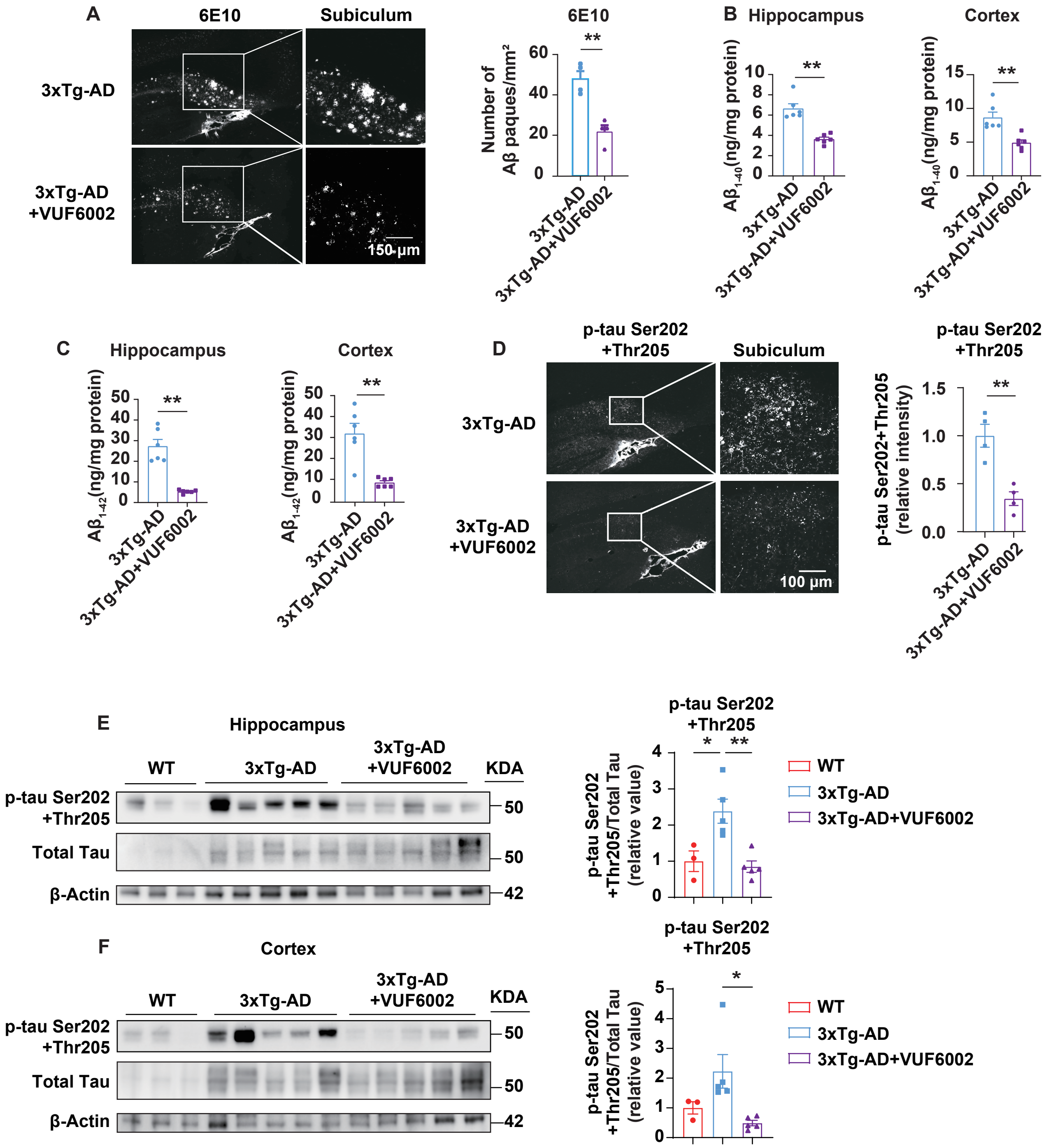

Fig. S9. VUF6002 alleviates Aβ and tau pathology in 15-month-old 3xTg-AD mice. (A) Representative confocal images showing a reduction in 6E10-positive Aβ plaques in the subiculum of 3xTg-AD mice following VUF6002 treatment. The number of Aβ plaques per mm² was quantified using NIS-Elements software (Nikon). Scale bar: 150 μm. (n = 4 per group). **(B-C)** The concentration of soluble human $A\beta_{1\text{-}40}$ and $A\beta_{1\text{-}42}$ in hippocampal and cortical lysates were measured by ELISA assay in vehicle- and VUF6002-treated 3xTg-AD mice (n = 6 per group). **(D)** Representative images showing that VUF6002 reduced phosphorylated tau (p-tau) at Ser202 and Thr205, as indicated by decreased fluorescence intensity in the subiculum of 3xTg-AD mice. Scale bar: 100 μm. **(E-F)** Western blot analysis demonstrated decreased levels of p-tau in the hippocampus (E) and cortex (F), normalized to Total Tau. Each lane represents a sample from a single mouse (n=3–5 per group). Mean ± SEM. * $P < 0.05$; ** $P < 0.01$; ns: not significant; one-way ANOVA followed by Tukey's post hoc test (A, and D–F). Student's t-test (B–C).

Figure S10

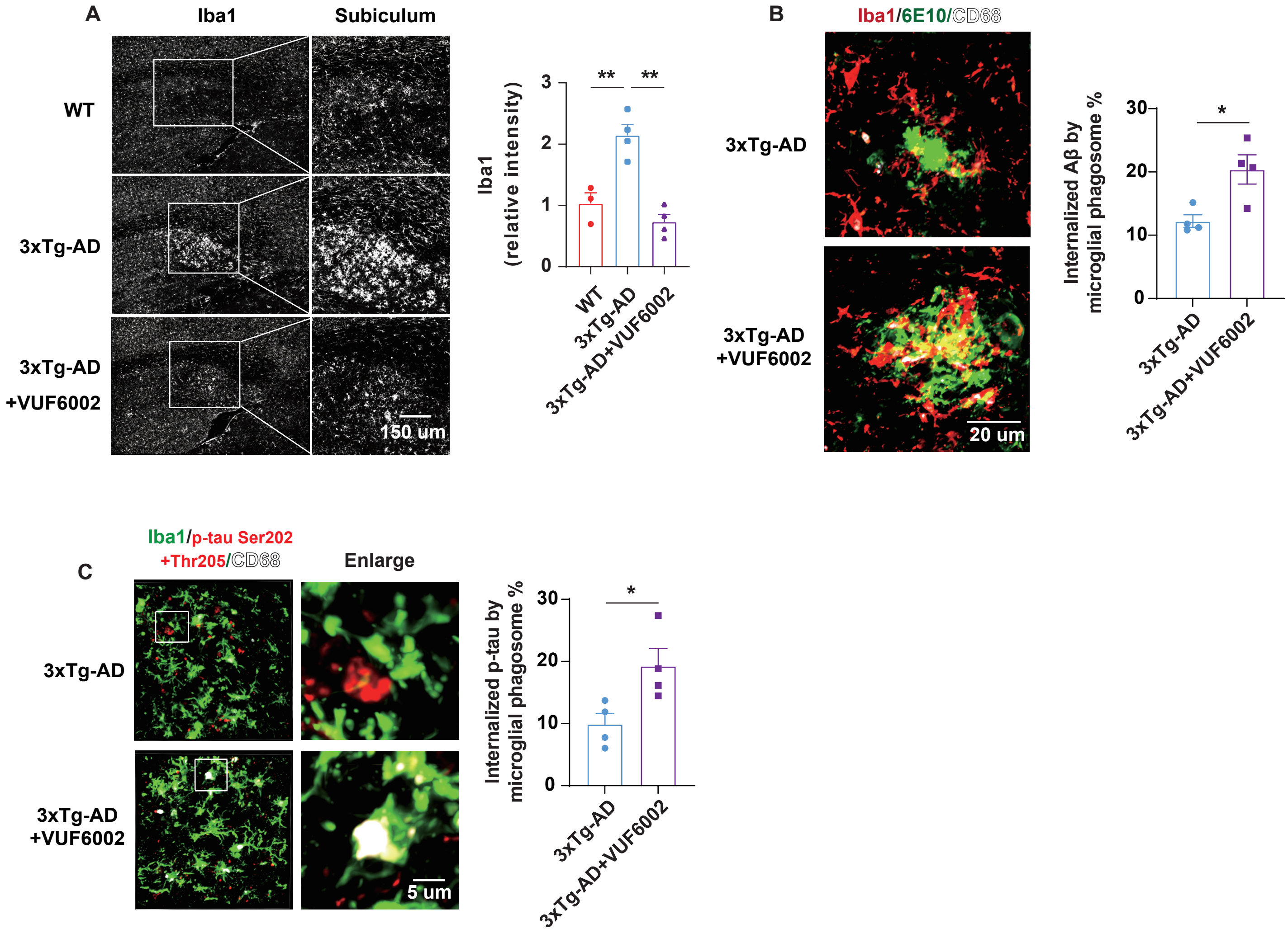

Fig. S10. VUF6002 inhibits microgliosis and promotes microglial phagocytic clearance of Aβ plaques in 15-month-old 3xTg-AD mice. (A) Representative confocal photomicrographs showing reduced microgliosis in the hippocampal subiculum region of VUF6002-treated 3xTg-AD mice. Scale bar: 150 μm. (n = 3–4 per group). **(B)** Three-dimensional (3D) reconstructions of microglia (Iba1, red), Aβ plaques (6E10, green) and lysosome (CD68, white) was quantified in the subiculum of aged 3xTg-AD mice. The colocalized volume of Iba1^{+}CD68^{+} lysosome with Aβ plaques was normalized to total plaques volume in vehicle- and VUF6002-treated mice (64 plaques per group). (n = 4 per group). **(C)** 3D reconstructions of microglia (Iba1, green), lysosomes (CD68, white), and phosphorylated tau (p-tau) Ser202+Thr205 (AT8, red) were analyzed in the subiculum. The colocalized volume of Iba1^{+}CD68^{+} lysosomes with p-tau was normalized to p-tau volume in aged 3xTg-AD mice and VUF6002-treated mice (64 images per group). (n = 4 per group). Mean ± SEM. * $P < 0.05$; ** $P < 0.01$, one-way ANOVA followed by Tukey's post hoc test (A); Student's t-test (B-C).

Figure S11

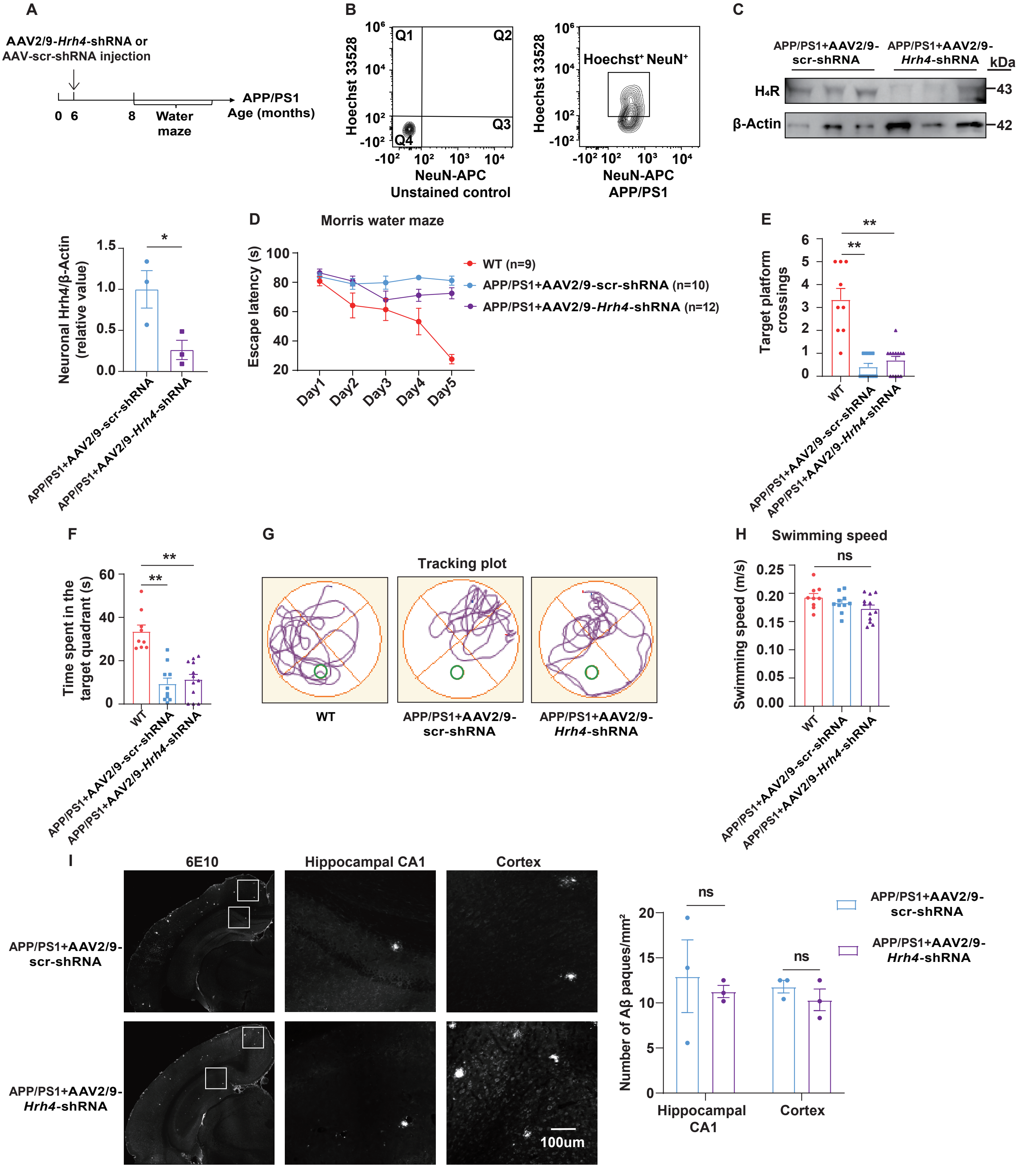

Fig. S11. Deletion of neuronal *Hrh4* does not affect cognitive functions or Aβ levels in 8-month-old APP/PS1 mice. (A) The 6-month-old APP/PS1 mice received intracranial injection of AAV2/9 vector expressing either a scramble control shRNA (AAV2/9-scr-shRNA) or a *Hrh4*-shRNA (AAV2/9-*Hrh4*-shRNA). Two months later, mice underwent Morris water maze testing to assess spatial memory. **(B)** A standard flow cytometry gating strategy was used to isolate neuronal cells from brain tissue. Hoechst 33528 stained nuclei, and NeuN antibody labeled neurons. Hoechst⁺/NeuN⁺ double-positive cells were sorted for downstream analysis. **(C)** Western blot quantification of FACS-sorted neurons showed a significant reduction in histamine H4 receptor protein levels in AAV2/9-*Hrh4*-shRNA injected mice compared to controls (AAV2/9-scr-shRNA). Each lane represents a sample from cortex and hippocampus, pooled from two mice. (n=6 per group). **(D-G)** Morris water maze results indicated no significant differences in latency (D), target platform crossings (E), or time spent in the target quadrant (F–G) between mice injected with scramble or *Hrh4*-shRNA. **(H)** Swimming speeds were also comparable across groups during the probe trial. (n = 9–12 per group). **(I)** Quantification of 6E10-positive Aβ plaque immunoreactivity showed no difference between the AAV2/9-scr-shRNA-treated and AAV2/9-*Hrh4*-shRNA treated mice. Scale bar: 100 μm. (n = 3 per group). Mean ± SEM, * $P < 0.05$; ** $P < 0.01$; ns: not significant; Student's t-test (C and I); two-way repeated-measures ANOVA followed by Tukey's post hoc test (D); one-way ANOVA followed by Tukey's post hoc test (E, F and H).

Figure S12

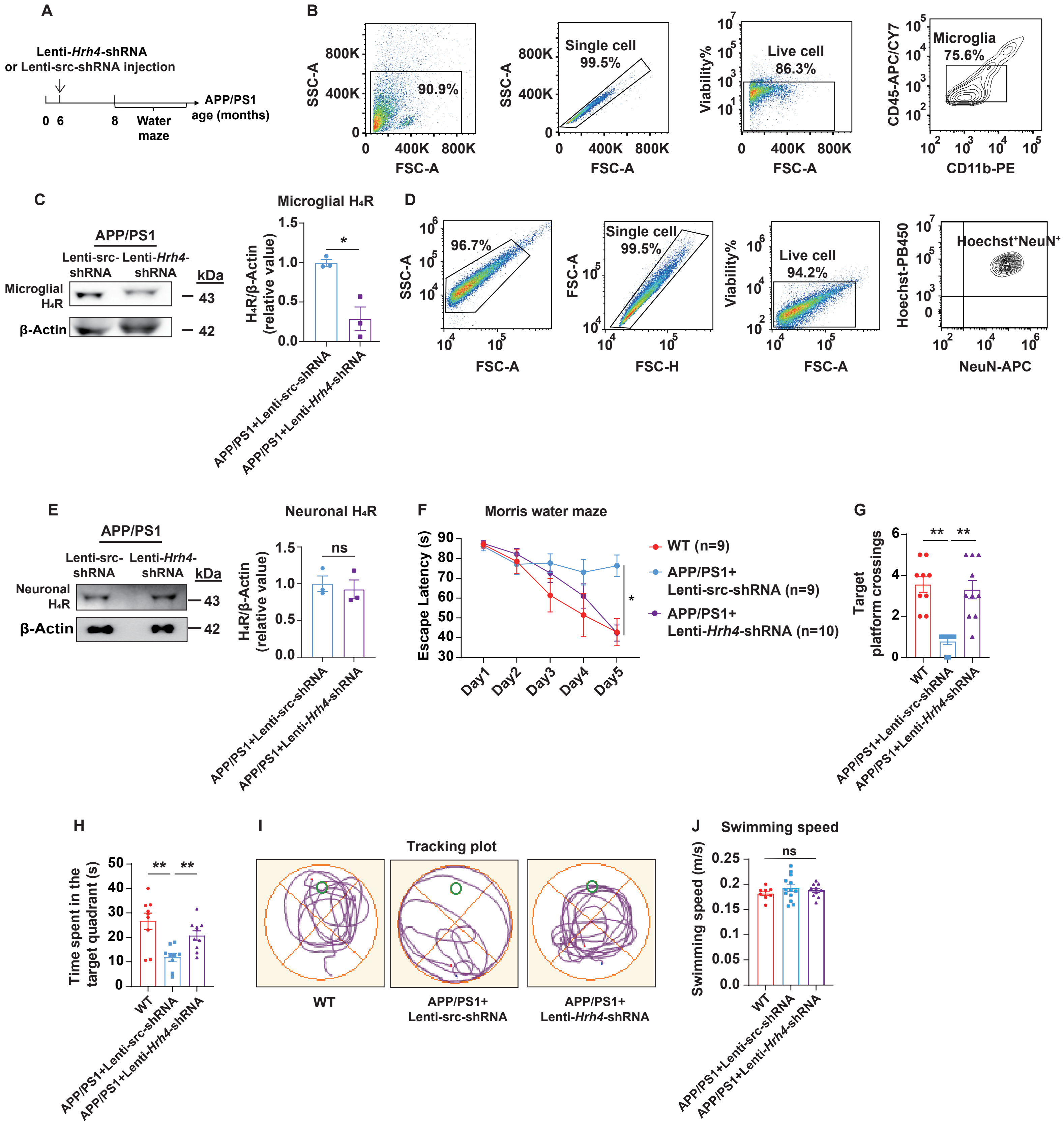

Fig. S12. Microglial deletion of *Hrh4* reverses the memory deficits in 8-month-old APP/PS1 mice. (A) The 6-month-old APP/PS1 mice received intracranial injection of lentivirus vector expressing either scramble control shRNA (Lenti-scr-shRNA) or shRNA targeting *Hrh4* (Lenti-*Hrh4*-shRNA) under a CX3CR1 promoter. **(B)** Standard flow cytometry gating strategy to sort microglia from the brain. CD11b positive and CD45 low expression cells ($CD11b^{+}CD45^{low}$) were sorted out as resident microglia for downstream analysis. **(C)** Western blot quantification of FACS-sorted microglia demonstrated a marked reduction in histamine H4 receptor (H_4R) protein levels (normalized to β-Actin) in Lenti-*Hrh4*-shRNA injected mice compared to scramble shRNA controls. Each lane represents a sample collected from the cortex and hippocampus, pooled from two mice. (n = 6 per group). **(D)** Standard flow cytometry gating strategy to sort the neurons in the brain. Hoechst 33528^{+} and NeuN^{+} double positive cells were sorted out as neurons. **(E)** The protein level of neuronal H_4R (normalized to β-Actin) was not affected by *Hrh4* knockdown (Lenti-*Hrh4*-shRNA). **(F)** APP/PS1 mice treated with shRNA for *Hrh4* exhibited comparable performance with age-matched wildtype (WT) control in finding the hidden platform. **(G-I)** In the probe test with the platform removed, the APP/PS1+Lenti-*Hrh4*-shRNA groups displayed comparable performance in the target platform crossings (G) and the time spent in the target quadrant (H-I) to WT mice level. **(J)** Swimming speeds revealed no difference across experimental groups. (n = 9 - 10 per group). Mean ± SEM, * $P < 0.05$; ** $P < 0.01$; ns: not significant; Student's t-test (C and E); two-way repeated-measures ANOVA followed by Tukey's post hoc test (F); one-way ANOVA followed by Tukey's post hoc test (G, H and J).

Figure S13

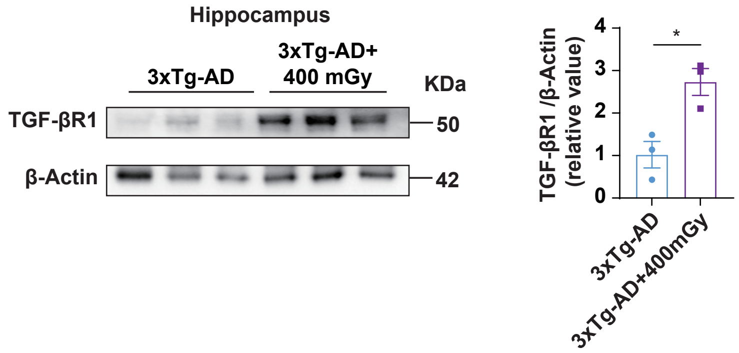

Fig. S13. LDIR activates TGF-β1 signaling in the hippocampus of aged 3xTg-AD mice. Western blot analysis showed a significant upregulation of TGF-βR1 levels in the hippocampus of LDIR-treated 3xTg-AD mice. TGF-βR1 expression was normalized to β-Actin. Each lane represents a sample from a single mouse. (n = 3 per group). Mean ± SEM, * $P < 0.05$; Student's t-test.

Figure S14

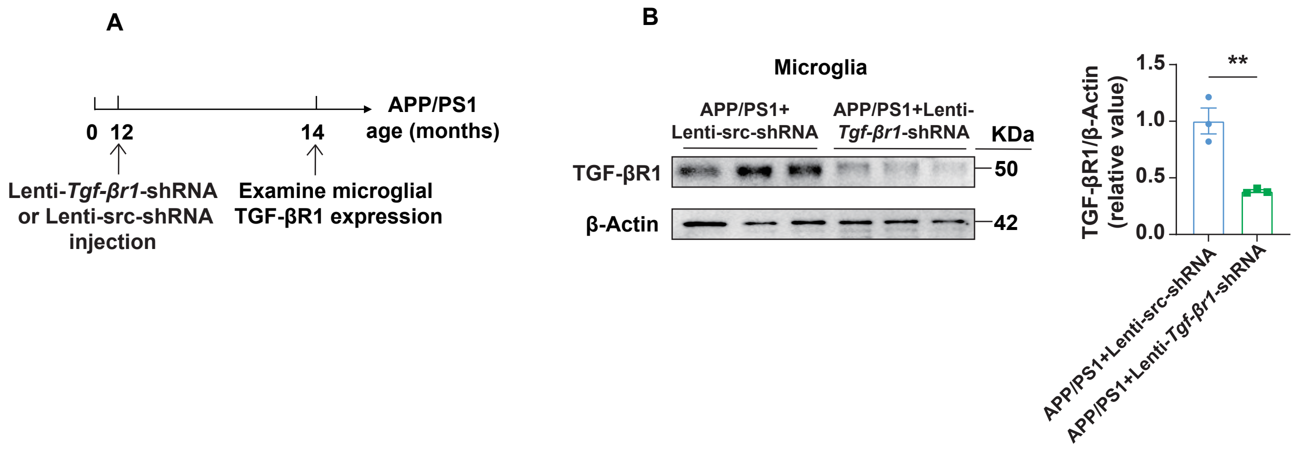

Fig. S14. Knockdown efficiency of microglial *Tgf-βr1* deletion. (A) The lentiviral vector carried shRNA targeting *Tgf-βr1* was injected to 12-month-old APP/PS1 mice. Two months post-injection, TGF-βR1 protein expression was examined in FACS-isolated microglia. **(B)** Western blot quantification of FACS-sorted microglia demonstrated a marked reduction in TGF-βR1 protein levels (normalized to β-Actin) in mice treated with Lenti-*Tgf-βr1*-shRNA compared to scramble shRNA controls. Each lane represents a sample from cortex and hippocampus tissue, pooled from two mice. (n = 6 per group). Mean ± SEM,, $^{**}P < 0.01$; Student t-test (B).

Figure S15

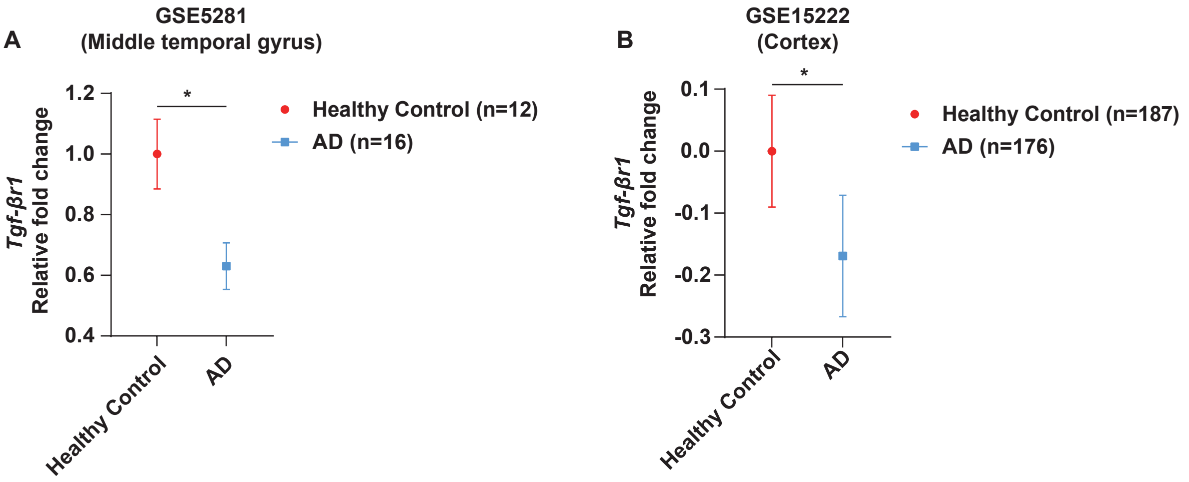

Fig. S15. Downregulation of *Tgf-βr1* expression in AD patients. (A-B) Transcriptional down-regulation of *Tgf-βr1* in (A) middle temporal gyrus of AD patients from GSE5281 database (Healthy Control, n = 12; AD patient, n = 16), and (B) cortex of AD patients from GSE15222 database (Healthy Control, n = 187; AD patient, n = 176). Mean ± SEM, ** $P < 0.01$, Mann–Whitney U test (A-B).
